# Glutathione Activates Type III Secretion System Through Vfr in *Pseudomonas aeruginosa*

**DOI:** 10.3389/fcimb.2019.00164

**Published:** 2019-05-16

**Authors:** Yani Zhang, Chao Zhang, Xiao Du, Yun Zhou, Weina Kong, Gee W. Lau, Gukui Chen, Gurjeet Singh Kohli, Liang Yang, Tietao Wang, Haihua Liang

**Affiliations:** ^1^Key Laboratory of Resource Biology and Biotechnology in Western China, College of Life Sciences, Northwest University, Ministry of Education, Xi'an, China; ^2^Department of Pathobiology, University of Illinois at Urbana-Champaign, Champaign, IL, United States; ^3^Singapore Centre for Environmental Life Sciences Engineering, Nanyang Technological University, Singapore, Singapore; ^4^Alfred Wegener-Institut Helmholtz-Zentrum für Polar- und Meeresforschung, Bremerhaven, Germany; ^5^School of Medicine, Southern University of Science and Technology, Shenzhen, China

**Keywords:** glutathione, type III secretion system, Vfr, pathogenicity, *Pseudomonas aeruginosa*

## Abstract

Glutathione (GSH) is the most abundant antioxidant in all living organisms. Previously, we have shown that a deletion mutant in the glutathione synthetase gene (Δ*gshB*) decreases the expression of type III secretion system (T3SS) genes of *Pseudomonas aeruginosa*. However, the mechanism remains elusive. In this study, a comprehensive transcriptomic analysis of the GSH-deficient mutant Δ*gshA*Δ*gshB* was used to elucidate the role of GSH in the pathogenesis of *P. aeruginosa*. The data show that the expression of genes in T3SS, type VI secretion system (T6SS) and some regulatory genes were impaired. Δ*gshA*Δ*gshB* was attenuated in a mouse model of acute pneumonia, swimming and swarming motilities, and biofilm formation. Under T3SS inducing conditions, GSH enhanced the expression of T3SS in both wild-type PAO1 and Δ*gshA*Δ*gshB*, but not in Δ*vfr*. Genetic complementation of Δ*vfr* restored the ability of GSH to induce the expression of T3SS genes. Site-directed mutagenesis based substitution of cysteine residues with alanine in Vfr protein abolished the induction of T3SS genes by GSH, confirming that GSH regulates T3SS genes through Vfr. Exposure to H_2_O_2_ decreased free thiol content on Vfr, indicating that the protein was sensitive to redox modification. Importantly, GSH restored the oxidized Vfr to reduced state. Collectively, these results suggest that GSH serves as an intracellular redox signal sensed by Vfr to upregulate T3SS expression in *P. aeruginos*a. Our work provides new insights into the role of GSH in *P. aeruginosa* pathogenesis.

## Introduction

*Pseudomonas aeruginosa* is a Gram-negative opportunistic pathogen that cause acute and chronic infections (Gellatly and Hancock, 2013). Protein secretion systems are essential for bacteria to intimately interface with other bacteria and the host, particularly in the efficiency of infection (Filloux, 2011). Large multicomponent secretion apparatus, including both type III secretion system (T3SS) and type VI secretion system (T6SS), facilitate *P. aeruginosa* infection by directly injecting effector proteins into the cytoplasm of host cells to subvert the host immune response. Four known T3SS effectors that have been identified are ExoS, ExoT, ExoY, and ExoU. Once injected into the host cells, these effectors disrupt cytoskeletal organization and host cell membranes, resulting in cytotoxicity and death (Brutinel et al., 2008).

The ExsA of *P. aeruginosa* is the central regulator of the of T3SS gene expression, and is encoded by the last gene of the *exsCEBA* polycistronic mRNA (Yahr and Wolfgang, 2006). In response to varieties of environmental signals (e.g., low Ca^2+^) and direct contact with eukaryotic host cells, ExsA autoregulates its own transcription through direct activation of the *exsC* promoter (Brutinel et al., 2008). Under non-inducing conditions (e.g., high Ca^2+^), ExsE and ExsC form a complex, allowing the anti-activators ExsD to bind to ExsA and inhibit transcription activation. Under inducible or low Ca^2+^ conditions, ExsE is secreted, which in turn, free ExsC to form a complex with ExsD, freeing ExsA to activate the expression of T3SS genes (King et al., 2012). Additionally, T3SS expression is also influenced by ~30 extrinsic gene products, including the Vfr (Diaz et al., 2011). Vfr is a member of the 3′, 5′-cAMP receptor protein that serves as a global transcriptional regulator of *P. aeruginosa* (Fuchs et al., 2010). Prior genomic and proteomic studies demonstrate that more than 100 genes and 60 proteins in *P. aeruginos*a are positively or negatively regulated by Vfr (Suh et al., 2002; Wolfgang et al., 2003). Importantly, a recent study showed that Vfr directly binds to the promoter of *exsA* and activates its transcription (Marsden et al., 2016).

Glutathione (GSH) is the most abundant intracellular thiol and antioxidant that protects cells against free radicals and reactive oxygen intermediates in living organisms. GSH is synthesized from three amino acid precursors L-glutamate, L-cysteine, and glycine by a two-step process involving the enzymes γ-glutamate-cysteine ligase (GshA) and GSH synthetase (GshB) (Lushchak, 2012). GshA catalyzes the ligation of cysteine with glutamate to form γ-glutamylcysteine. GshB ligates γ-glutamylcysteine with glycine to generate glutathione. Previously, we have shown that *P. aeruginosa* mutant strain deficient in GshB expresses lower level of T3SS (Zhang and Wei, 2009). Additionally, GSH could alter *P. aeruginosa's* sensitivity to different antibiotics (Zhang and Duan, 2009). In this study, we discover that the GSH biosynthesis deficient mutant Δ*gshA*Δ*gshB* expresses significantly lower level of T3SS. Further study supports a model in which the global regulator Vfr senses GSH and activates the expression of T3SS in *P. aeruginos*a.

## Materials and Methods

### Bacterial Strains and Growth Conditions

All bacterial strains, plasmids and primers used in this study are listed in Tables S1, S2. Bacteria were maintained in Luria Bertani (LB) broth or Pseudomonas Isolation Agar at 37°C. Antibiotics were added to the growth medium as needed: for *E. coli*: 15 μg/ml gentamicin (Gm), 50 μg/ml kanamycin (Kan), 100 μg/ml ampicillin (Amp), and 10 μg/ml tetracycline (Tet) in LB medium; For *P. aeruginosa* strain PAO1: 50 μg/ml Gm in LB medium or 150 μg/ml in Pseudomonas isolation agar (PIA), and 300 μg/ml trimethoprim (Tmp) and 500 μg/ml carbenicillin (Cb) in LB medium. Depletion of intracellular GSH in *P. aeruginosa* was performed with 1.5 mM diethylmaleate (DEM). Biofilm formation in flow chamber was performed in ABTG medium (1.51 mM (NH_4_)_2_SO_4_, 3.37 mM Na_2_HPO_4_, 2.2 mM KH_2_PO_4_, 5 mM NaCl, 1 mM MgCl_2_·6H_2_O, 100 μM CaCl_2_·2H_2_O, 1 μM FeCl_3_·6H_2_O, and supplemented with 0.4 g/l Glucose).

### Construction of Plasmids and Deletion Mutants

The Δ*gshB* was as previously described (Zhang and Duan, 2009) (Table S1). For deletion of the *gshA* gene, a 1,948 bp fragment upstream of the start codon and a 1,966 bp fragment downstream of the stop codon flanking the gene were PCR amplified and digested with *Hin*dIII-*Xba*I and *Xba*I-*Eco*RI, respectively. The two fragments were then ligated into pEX18Tc plasmid, which was digested with *EcoR*I and *Hin*dIII, resulting in pEX18Tc*-gshA*, in which the whole *gshA* open reading frame (ORF) had been deleted. The resultant plasmid was electroporated into PAO1, followed by selection for single crossovers and double crossovers as described previously (Kong et al., 2015). Both Δ*exsA* and Δ*vfr* were generated by using a similar strategy. Deletions of the *gshA* gene in Δ*gshB* mutant strain, and the *vfr* gene in Δ*gshA*Δ*gshB* mutant strain were constructed using similar strategy. Successful deletions were verified by PCR.

To genetically complement the Δ*gshB* mutant, the promoter and coding regions of the *gshB* locus was PCR amplified from PAO1 genomic DNA using primers listed on Table S2, and integrated into Δ*gshB* genome at the *attB* site using the mini-CTX1 system (Hoang et al., 2000). The result was confirmed by PCR amplification. The complemented strain was named Δ*gshB*/*gshB*.

To genetically complement the Δ*vfr*, a 916 kb fragment containing the *vfr* coding regions with its own promoter was PCR amplified from PAO1 genomic DNA using primers listed in Table S2, digested with *Bam*HI and *Hin*dIII, and then ligated into a shuttle vector, pAK1900 (Poole et al., 1993), resulting in pAK-*vfr*. pAK-*gshB* was constructed using the similar strategy.

The plasmid pMS402 with a promoterless *luxABCDE* gene cluster was used to construct the *lux* reporter strains (Duan et al., 2003). For examples, the *exoS* promoter region were amplified by PCR and cloned into the pMS402, yielding pKD-*exoS*. Similar strategy was used to clone the promoters of *exoY, exsA, exsC*, and *vfr* in front of the promoterless *lux*, respectively. Apart from the plasmid-based *lux* reporter system, an integration plasmid CTX 6.1 derived from the plasmid mini-CTX-*lux* was used to construct the chromosomal fusion reporter (Becher and Schweizer, 2000). The pMS402 fragment containing the MCS kanamycin-resistance marker and the promoter-lux reporter cassette were ligated into CTX 6.1, yielding CTX-promoter-*lu*x and electroporated into *P. aeruginosa* recipient strains. All constructs were sequenced to verify that no mutations had been incorporated.

### H_2_O_2_ Susceptibility Measurements

For the agar diffusion assay, *P. aeruginosa* strains were cultured overnight in LB broth and adjusted to an optical density OD_600_ of 0.1, then, 20 μl of bacteria was suspended in 30 ml of LB soft agar (0.7% agar), mixed, and incubated on agar plates for 30 min. Then, 5 μl of 30% H_2_O_2_ was spotted onto each disk prepopulated on the agar plates containing the bacteria. After overnight incubation at 37°C, inhibition zones were measured. All experiments were performed in triplicate.

### Growth Studies

*P. aeruginosa* strains were cultivated in LB medium overnight and then diluted 1:100 in fresh media and grown in 96-well plates with shaking at 37°C. Bacterial growth (OD_600_) was measured every 2 h for 24 h and each strain was assayed in triplicate.

### Gene-Expression Assays

The level of gene expression was monitored by measuring luminescence production in black 96-well plates with transparent bottoms. Both luminescence (counts per second) and bacterial growth (OD_595_) were measured every 30 min for 24 h in a Victor^2^ Multilabel Counter (Perkin-Elmer) as previously described (Zhang et al., 2017).

### Swimming, Swarming, and Twitching Motility Assays

Swimming and swarming motilities were assayed as described (Rashid and Kornberg, 2000). Swimming media consisting of 0.3% agar supplemented with 1% tryptone and 0.5% NaCl. Swarming media consisting of 0.4% agar supplemented with 0.8% nutrient broth and 0.5% glucose. Twitching medium consisting of 1% agar supplemented with 1% tryptone, 0.5% yeast extract and 1% NaCl. *P. aeruginosa* cultures were diluted to an OD_600_ of 0.1, and 2 μl of the diluted bacteria was center-spotted onto the surface of the agar plates. After the absorption of spotted bacterial inoculum, swimming plates were incubated at 30°C for 14–16 h, while swarming and twitching plates were incubated at 37°C for 14–16 h. Bacterial motility was imaged using a camera (LAS-3000, Tanon, China).

### Biofilm Formation Assays

Biofilm formation in LB was performed as previously described (O'Toole and Kolter, 1998) with minor modifications. Briefly, 800 μl of the diluted bacterial suspension (OD_600_ = 0.1) was incubated in 24-well polystyrene plates. Three milliliters of the diluted bacteria were also incubated in borosilicate tubes to visualize biofilms. After 30 h of incubation at 37°C without shaking, planktonic bacterial cultures were gently removed. The plates and tubes were triple-washed with distilled water, and the adhered bacteria were stained with 0.1% crystal violet for 20 min. Then, the plates and tubes were washed with water to remove unbound dye and air dried. To quantify the biofilms on the 24-well polystyrene plate, 800 μl of 95% ethanol was added to each well to elute the crystal violet. The absorbance of the pooled eluent was measured at 590 nm by a microplate reader (Infinite M200 PRO, TECAN, Switzerland). Experiments were performed in triplicate, and results are shown as the mean ± s.d.

Development of biofilms in a flow chamber were performed as previously described (Chua et al., 2016). Various *gfp*-tagged *P. aeruginosa* strains were cultured overnight in 2 ml of LB medium in 37°C with shaking condition (200 rpm). The overnight cultures were diluted to 1:100 with ABTG medium and 500 μl of the diluted cultures were injected into the flow chambers and incubated for 1 h incubation in an inverted position without medium flow (Sternberg and Tolker-Nielsen, 2006). Then, the flow chambers were placed in upright position before the bacteria were fed with ABTG medium via Masterflex peristaltic pump (Cole-Parmer, United States) at the rate of 4 ml/h in 37°C to allow biofilm development. After 72 h, 300 μl of 3.34 μM SYTO9 (LIVE/DEAD™ BacLight™ Bacterial Viability Kit, ThermoFisher Scientific) was injected into each chamber to stain the bacteria for 15 min before subjected to confocal microscopy. The images of the SYTO9-stained biofilms were acquired using an LSM 780 confocal laser scanning microscope (Carl Zeiss, Germany) fitted with 20×/0.8 DICII objective lens and 488 nm argon multiline laser. Acquired images were further processed using the IMARIS Version 8 software (Bitplane AG, Zurich, Switzerland) to obtain the volume of biofilms.

### Mouse Model of Acute Pneumonia Infection

All animal experiments were performed in strict accordance with the Regulations for the Administration of Affairs Concerning Experimental Animals approved by the State Council of People's Republic of China (11-14-1988). All animal procedures were approved by the Institutional Animal Care and Use Committee (IACUC) of the college of life sciences of Northwest University with a permit number: NW-02-2014. Acute lung infection was performed as described previously (Li et al., 2014). Briefly, stationary phase *P. aeruginosa* grown in LB were harvested, washed three times, and serially diluted in PBS and infected intranasally (1 × 10^7^ CFU) into the 6-week old male CD-1 mice. For bacterial burden determination, infected mice (*n* = 6) were euthanized at 16 h post-infection. Mouse lungs were aseptically removed and homogenized in 1 ml cold PBS with a Dounce homogenizer. Homogenates were serial diluted and plated on *Pseudomonas* isolation agar. Colony forming units (CFU) were determined after 24 h of incubation at 37°C. For histopathological analyses, lungs were inflated with 1 ml 10% neutral buffered formalin, harvested and immersed in the same solution for 24 h prior to paraffin and sectioning. Paraffin embedded sections (4- to 8-mm thickness) were stained with hematoxylin and eosin (H&E).

### RNA-seq and Data Analyses

Identification of the GSH regulons in *P. aeruginosa* genome by RNA-seq was performed as previously described (Deng et al., 2014). Briefly, 10 ml of mid-log-phase of PAO1 and Δ*gshA*Δ*gshB* strains were collected. An RNeasy minikit (Qiagen) was used for RNA purification with DNase I treatment. After removing rRNA by using the MICROBExpress kit (Ambion), mRNA was used to generate the cDNA library according to the TruSeq RNA sample prep kit protocol (Illumina), and sequenced using the HiSeq 2000 System. Bacterial RNA-seq reads were mapped to the *P. aeruginosa* genomes by using TopHat (version 2.0.0), with two mismatches allowed. Only the uniquely mapped reads were kept for subsequent analyses. The analysis of differentially expressed genes was performed using Cuffdiff software (version 2.0.0). The data have been uploaded to NCBI under BioProject (accession number PRJNA504392).

### RNA Extraction and Quantitative Real-Time PCR

Overnight cultures of *P. aeruginosa* were diluted 1:100 with fresh LB broth and recultured until OD_600_ 0.6. RNA was isolated from bacteria using the RNAprep pure cell/bacteria Kit (Tiangen Biotech, Beijing, China). cDNA was synthesized using the PrimeScript Reverse Transcriptase (TaKaRa) with random primers, then subjected to qRT-PCR using the SYBR Premix Ex Taq II (TaKaRa). The 30s ribosomal protein gene *rpsL* was used as an internal control. Data presented were averages of three independent cultures grown on different days.

### Western Blot Assays

The production of ExoS was analyzed as previously described (Bleves et al., 2005). Overnight *P. aeruginosa* cultures were subcultured with 100-fod dilution in fresh LB containing 5 mM EGTA and 20 mM MgCl_2_ for 3 h. Bacterial supernatant and pellet were separated by centrifugation. Bacterial supernatant was treated with 15% trichloroacetic acid (TCA) at 4°C to precipitate proteins. Precipitated proteins were collected by centrifugation and washed with acetone. Then, protein samples from equivalent numbers of bacterial cells were loaded and separated on 12% SDS-PAGE gels. Proteins were transferred onto a polyvinylidene difluoride (PVDF) membrane and hybridized with a rabbit polyclonal antibody against ExoS generously provided by Professor Weihui Wu (Nankai University). The signal was detected by an ECL Plus kit (Amersham Biosciences). All Western blot assays were repeated a minimum of three times with independently derived proteins samples. Representative blots are shown.

### Site-Directed Mutagenesis

Alanine (GCT) substitution of Cys20, Cys38, Cys97, Cys156, and Cys183 in Vfr was performed by using a site-directed mutagenesis kit (TransGen Biotech). All mutagenesis primers are listed in Table S2.

### Protein Purification of Vfr

A *P. aeruginosa vfr* expression plasmid ptac-*vfr* (Table S1) was introduced into *P. aeruginosa* PA103 for Vfr purification as described, with modifications (Dasgupta et al., 2002). Briefly, cultures were grown to the density of OD_600_ 0.6, and then isopropyl-β-D-thiogalactopyranoside (IPTG) was added to a final concentration of 1 mM to induce Vfr expression. Following an additional 7 h incubation at 37°C, bacteria were harvested by centrifugation at 10,000 × g for 4 min at 4°C. The cell pellet was resuspended in buffer A (80 mM KCl, 50 mM K_3_PO_4_, pH 7.2) with 10 mM PMSF at a ratio of 5 ml/g of cells (wet weight) and lysed by sonication in an ice bath. Lysates were centrifuged (11,000 rpm for 60 min at 4°C) to remove unbroken cells and insoluble materials. All subsequent steps were performed at 4°C. Soluble materials were filtered through a 0.45 μm filter and applied to a HiTrap HP column (GE Healthcare) equilibrated with buffer A. The column was washed with 10% buffer B (1M KCl, 50 mM K_3_PO_4_, pH 7.2) and eluted with a linear gradient from 10% to 100% buffer B over 40 ml using an ÄKTA Prime. Peak fractions were collected and enriched, and then load onto a Gel Filtration column (GE Healthcare) equilibrated with buffer A. Vfr-containing fractions were identified by SDS-PAGE, pooled and stored at −80°C until further use.

### Detection Thiol Status of Vfr Protein With AMS

Cysteine thiols in protein were irreversibly modified with 4-acetamido-4′-maleiniDystil-bene-2,2′-disufonic acid (AMS), which adds 0.5 kDa mass to every modified cysteine (Si et al., 2015). The increase in protein mass was evident as delay of electrophoretic mobility of AMS-labeled proteins. Briefly, Vfr proteins were incubated with or without 1 mM DTT at room temperature for 30 min, and excess DTT was removed by ultrafiltration. GSH (15 mM) and H_2_O_2_ (500 mM) treated protein were analyzed with same strategy. The resulting proteins were incubated with 30 mM AMS for 30 min and then separated by non-reducing SDS-PAGE.

### Circular Dichroism Spectroscopy Studies

CD measurements were performed by using a spectrometer (Chirascan V100, Applied Photophysics, England) equipped with a 1 mm path length quartz cell according to a method described previously (Kessenbrock and Groth, 2017). The CD spectra were recorded in the range from 190 to 260 nm. The helical content of protein was calculated based on change in molar ellipticity value.

### Statistical Analysis

All experiments were performed in triplicate and independently repeated three times. Data were analyzed using an Unpaired Student's *t*-test and ANOVA, and expressed as the mean ± standard deviation. The differences between means were considered be significant at *p* < 0.05.

## Results

### Δ*gshB* and Δ*gshA*Δ*gshB* Mutant Exhibits Motility and Biofilm Formation Defects

Our previous study revealed that GSH alters the sensitivity of *P. aeruginosa* to different antibiotics, as well as modulates the expression of T3SS genes (Zhang and Duan, 2009; Zhang and Wei, 2009). Reduction in the expression of T3SS may reduce the virulence of GSH-deficient *P. aeruginosa* mutant. *gshA* and *gshB* are responsible for GSH biosynthesis in *P. aeruginosa*. In other bacteria, such as *E. coli* and *L. monocytogenes*, Δ*gshB* strain lacks reduced GSH but instead accumulates the glutathione precursor compound γ-glutamylcysteine which partially functionally replace GSH (Fuchs and Warner, 1975; Gopal et al., 2005). Here, to investigate the role of GSH in *P. aeruginosa*, γ-glutamate-cysteine ligase mutant (Δ*gshA*) and double *gshA gshB* mutant (Δ*gshAgshB*) were constructed. Δ*gshAgshB* strain lacking both glutathione and γ-glutamylcysteine. When cultured in LB broth, Δ*gshA*, Δ*gshB*, and Δ*gshA*Δ*gshB* mutant strains showed similar growth kinetics as PAO1 (Figure S1A). Because GSH is a major intracellular antioxidant protecting against environmental stresses, we assessed the susceptibility of these mutants to H_2_O_2_ *in vitro*. When compared to PAO1, Δ*gshB* and Δ*gshA*Δ*gshB* were more susceptible to H_2_O_2_, while Δ*gshAgshB* mutant completely lacking GSH described greater susceptibility to H_2_O_2_ than Δ*gshB* mutant (Figure S1B; Table S4), confirming the protective function of GSH against oxidative stress and injury.

**Figure 1:**
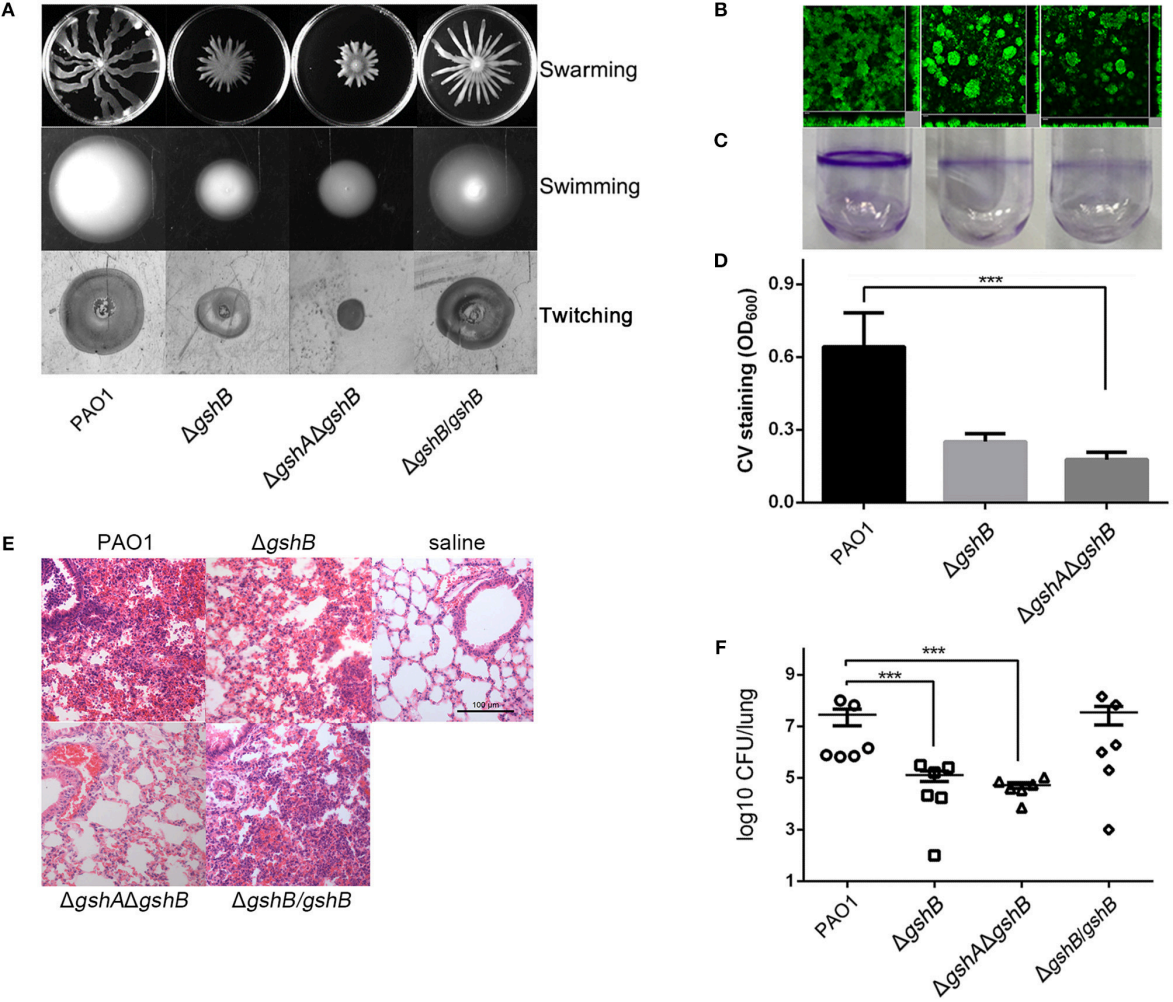
GSH is important in *P. aeruginosa* pathogenicity. **(A)** Δ*gshB* and Δ*gshA*Δ*gshB* exhibited weaker swimming, swarming, and twitching motilities. The experiments were repeated at least three times with similar results. Images from one typical experiment are shown. **(B)** Biofilms of PAO1, Δ*gshB* and Δ*gshA*Δ*gshB* grown in flow cells for 72 h and examined by confocal laser scanning microscopy. Experiments were performed in triplicate independently for three times. Representative images are shown. **(C,D)** Biofilm formation of PAO1, Δ*gshB* and Δ*gshA*Δ*gshB* on borosilicate tube surface and quantified by the crystal violet (CV) staining methods. The results are averages based on three replicate tubes. **(E)** H&E stained mouse lungs 16 h post-infection by indicated *P. aeruginosa* strains. **(F)** Δ*gshB* and Δ*gshA*Δ*gshB* are attenuated in acute pneumonia infection. Bacterial burden in CD-1 mouse lungs (*n* = 6) after 16 h of infection by various *P. aeruginos*a strains. Data represent average CFU/lung ± standard deviations. ***p < 0.001, when compared PAO1 to Δ*gshB* and Δ*gshA*Δ*gshB*.

To determine the effects of GSH on *P. aeruginosa* pathogenesis, we compared the motility and biofilm of Δ*gshB* and Δ*gshA*Δ*gshB* against the wild-type PAO1. Both Δ*gshB* and Δ*gshA*Δ*gshB* were attenuated in swarming, swimming and twitching motility. The genetically complemented strain Δ*gshB*/*gshB* showed wild-type levels of motilities, suggesting that defects in motility were due to the lack of GSH (Figure 1A).

Biofilm formation is partially dependent on bacterial swarming motility (Shrout et al., 2006; Pamp and Tolker-Nielsen, 2007; Chow et al., 2011). We examined whether GSH deficiency impacted the ability of Δ*gshB* and Δ*gshA*Δ*gshB* to produce biofilms. In the flow cell system, Δ*gshB* and Δ*gshA*Δ*gshB* synthesized significantly lower amounts of biofilms than wild-type PAO1 (Figure 1B). Similarly, the crystal violet method revealed that biofilms formed by Δ*gshB* and Δ*gshA*Δ*gshB* were between 2- and 3-fold lower than PAO1 (Figures 1C,D). These results suggest that the loss of GSH biosynthesis impairs both motility and biofilm formation of *P. aeruginosa*.

### Δ*gshB* and Δ*gshA*Δ*gshB* Are Attenuated in a Mouse Model of Acute Pneumonia

Defective motility and biofilm formation in Δ*gshB* and Δ*gshA*Δ*gshB* suggest that GSH modulates *P. aeruginosa* pathogenicity. Therefore, we investigated whether both Δ*gshB* and Δ*gshA*Δ*gshB* were attenuated in lung infection. As shown in Figure 1E, when compared to the saline vehicle control group, PAO1-infected lungs showed more severe inflammation with intense infiltration of proinflammatory cells and hemorrhaging involving small bronchi, bronchioles, and alveoli. In contrast, inflammatory cell recruitment was greatly reduced in mouse lungs infected with both Δ*gshB* and Δ*gshA*Δ*gshB*. Additionally, the Δ*gshA*Δ*gshB* mutant induced less severe pneumonia than the Δ*gshB*. Similar to the histopathological observation, the bacterial burden in mouse lungs infected with PAO1 were significantly higher than those infected by Δ*gshB* or Δ*gshA*Δ*gshB*, with a median value of 2.8 × 10^7^ CFU/lung. Conversely, the average bacterial burden in mice infected with Δ*gshB* and Δ*gshA*Δ*gshB* was approximately 100-fold and 200-fold lower, at 1.27 × 10^5^ and 5.24 × 10^4^ CFU/lung, respectively (Figure 1F). Genetic complementation of Δ*gshB* (Δ*gshB/gshB*) restored the lost virulence. Finally, **Δ***gshA* was also attenuated in mouse model of acute pneumonia (Figure S1C). Collectively, these results indicate that GSH plays crucial role in conferring full virulence in *P. aeruginosa*.

### Transcriptomic Analysis of the Δ*gshA*Δ*gshB*

**Figure 2:**
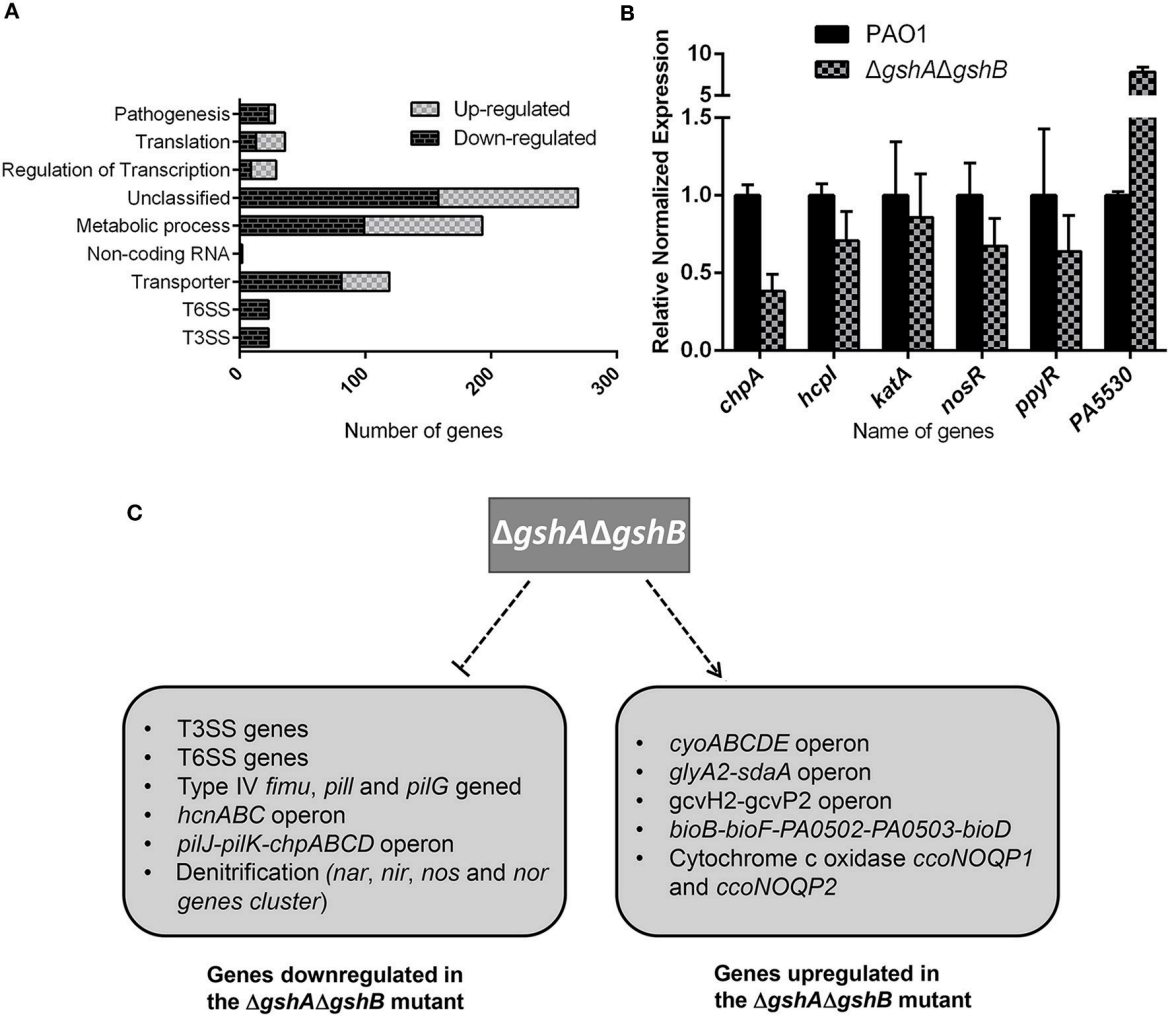
Comparison of the expression of selective genes between wild type PAO1 and Δ*gshA*Δ*gshB*. **(A)** Functional categorization of differentially expressed genes according to their annotated function. Numbers of genes whose expression were altered in Δ*gshA*Δ*gshB* compared to PAO1 were shown. **(B)** RT-PCR analysis of selective genes in PAO1 vs. Δ*gshA*Δ*gshB*. All data shown were means ± SEM. RT-PCR assays were performed in triplicate. **(C)** Summary of major gene categories upregulated or repressed by GSH in *P. aeruginosa*.

In attempt to have a comprehensive gauge of the function of GSH in *P. aeruginosa*, we compared the transcriptome of Δ*gshA*Δ*gshB* to its parental wild-type PAO1 by RNA-seq analysis. A total of 676 genes with altered transcript levels were identified, with 294 genes showing an increase and 382 genes showing a decrease of least 2-fold (i.e., log_2_ = 2) (Table S3). The *gshA/gshB* deficiency altered the expression of numerous genes coding for proteins involved in regulatory functions, pathogenesis, translation, transporter, T3SS, T6SS, and metabolism (Figure 2A). To assess the reliability of the RNA-seq approach, we used the identical total RNA samples and determined by RT-qPCR the mRNA levels of one upregulated gene (PA5530) and five downregulated genes (*chpA, hcp1, katA, nosR, ppyR*). Expression levels of these genes closely resembled those obtained from the RNA-seq experiment (Figure 2B).

Differentially expressed genes were classified into functional categories according to the PseudoCAP designations and information from other studies. Among the 676 genes, expression of those genes involved in motility, T3SS, T6SS, secreted factors (toxins, enzymes and alginate), and denitrification *(nar, nir, nos*, and *nor*) were repressed whereas expression of genes involved in quinol oxidase (*cyo* cluster), cytochrome c oxidases (*ccoNOQP1* and *ccoNOQP2*), and amino acid biosynthesis were induced (Filiatrault et al., 2005) (Figure 2C). The *nar, nir*, and *nos* genes are required for anaerobic growth. In contrast, the *cyo* cluster, *ccoNOQP1*, and *ccoNOQP2* are required for aerobic growth (Wu et al., 2005; Frangipani et al., 2008). Quinol oxidase operon and cytochrome c oxidases function as a low affinity terminal oxidase under high-oxygen, aerobic respiration conditions (Frangipani et al., 2008). These results suggest GSH modulates the expression of genes involved in respiratory pathways in *P. aeruginosa*. Importantly, the expression of 27 genes in T3SS secretion system, including *exsA, exsD, exoY, exoS, pcrD, pcrG, pcrV, pscS, pscP, pscQ*, and *pscBCDEFGHIJ* was reduced in Δ*gshA*Δ*gshB* when compared to wild-type strain. Similarly, 28 genes involved in T6SS secretion were repressed in Δ*gshA*Δ*gshB* (range from 2- to 6.5-fold), including both Hcp1 secretion island I (HSI-1) (PA1656-PA1690) and HSI-II (*icmF1, ppkA*, PA0086-PA0091) (Chen et al., 2015) (Table 1). Collectively, these results suggested that GSH regulates the expression of numerous virulence genes in *P. aeruginosa*. For the remainder of this study, we performed detailed analysis of the regulation of T3SS by GSH.

**Table 1:** Genes with altered expression in Δ*gshA*Δ*gshB* when compared to parental wild-type PAO1.

**Locus tag**	**Gene name**	**Fold change**	**Gene function**
**T3SS GENES**			
PA0044	*exoT*	−4.45466758	exoenzyme T
PA 1689	PA1689	2.071591324	Conserved hypothetical protein in T3SS
PA 1694	*pscQ*	−2.35035367	Translocation protein in T3SS secretion
PA 1695	*pscP*	−2.19640736	Translocation protein in T3SS secretion
PA 1701	*pcr3*	−2.363138	Regulatory protein in T3SS
PA 1703	*pcrD*	−2.55773203	T3SS secretory apparatus protein PcrD
PA 1704	*pcrR*	−3.75650633	Transcriptional regulator protein PcrR
PA 1705	*pcrG*	−3.18167213	Regulator in T3SS secretion
PA1706	*pcrV*	−3.38478764	T3SS secretion protein PcrV
PA1707	*pcrH*	−5.92843804	Regulatory protein PcrH
PA1708	*popB*	−4.78755524	Translocator protein PopB
PA1709	*popD*	−4.43846198	Translocator outer membrane protein PopD precursor
PA1710	*exsC*	−2.75707595	ExsC, exoenzyme S synthesis protein C precursor
PA1711	*exsE*	−4.08813812	Regulatory protein ExsE in T3SS
PA1712	*exsB*	−2.46600588	Exoenzyme S synthesis protein B
PA1713	*exsA*	−2.57563098	Transcriptional regulator ExsA
PA1714	*exsD*	−2.20197725	Regulatory protein ExsD in T3SS
PA1715	*pscB*	−2.26345008	T3SS export apparatus protein
PA1716	*pscC*	−2.60924525	T3SS secretion outer membrane protein PscC precursor
PA1717	*pscD*	−3.02363447	T3SS export protein PscD
PA1718	*pscE*	−2.90851002	T3SS export protein PscE
PA1719	*pscF*	−2.56778518	T3SS export protein PscF
PA1720	*pscG*	−3.62091142	T3SS export protein PscG
PA1721	*pscH*	−2.2775302	T3SS export protein PscH
PA1722	*pscI*	−2.43668146	T3SS export protein PscI
PA1723	*pscJ*	−2.16915564	T3SS export protein PscJ
PA2191	*exoY*	−3.28248108	Adenylate cyclase ExoY
**T6SS GENES**			
PA0074	*ppkA*	−2.11898918	Serine/threonine protein kinase PpkA
PA0077	*icmF1*	−6.31173974	Export apparatus IcmF1 in T6SS
PA0078	*tssL1*	−2.15686054	Export apparatusTssL1 in T6SS
PA0083	*tssB1*	−6.21885291	Protein secretion/export apparatus in T6SS
PA0084	*tssC1*	−5.92072778	Protein secretion/export apparatus in T6SS
PA0085	*hcp1*	−3.77657278	Secreted Factors Hcp1 in T6SS
PA0086	*tagJ1*	−2.14834322	Secretion protein in T6SS
PA0087	*tssE1*	−5.52513332	Secretion protein in T6SS
PA0088	*tssF1*	−4.8468817	Secretion protein in T6SS
PA0089	*tssG1*	−4.6167897	Secretion protein in T6SS
PA0090	*clpV1*	−3.68195792	ClpA/B–type chaperone
PA0091	*vgrG1*	−4.77451624	VgrG1 in T6SS
PA1656	*hsiA2*	−5.30038961	Protein secretion/export apparatus
PA1657	*hsiB2*	−2.06590215	Protein secretion/export apparatus
PA1658	*hsiC2*	−2.42591207	Protein secretion/export apparatus
PA1659	*hsiF2*	−2.82575912	Protein secretion/export apparatus
PA1660	*hsiG2*	−3.8839074	Protein secretion/export apparatus
PA1661	*hsiH2*	−4.41376322	Protein secretion/export apparatus
PA1662	*clpV2*	−2.01011682	Protein secretion/export apparatus
PA1663	*sfa2*	−3.1571465	Protein secretion/export apparatus
PA1664	*orfX*	−4.14296746	Protein secretion/export apparatus
PA1665	*fha2*	−2.50737402	Protein secretion/export apparatus
PA1666	*lip2*	−2.51739551	Protein secretion/export apparatus
PA1667	*hsiJ2*	−4.52360165	Protein secretion/export apparatus
PA1668	*dotU2*	−2.53779029	Protein secretion/export apparatus
PA1669	*icmF2*	−2.71554212	Protein secretion/export apparatus
PA1670	*stp1*	−4.28588287	Protein secretion/export apparatus
PA1671	*stk1*	−5.62977334	Protein secretion/export apparatus
**TYPE 4 PILI BIOGENESIS GENES**			
PA0410	*pilI*	−7.94797102	Twitching motility protein PilI
PA0411	*pilJ*	−10.0863067	Twitching motility protein PilJ
PA0412	*pilK*	−9.58293728	Methyltransferase PilK
PA0413	*chpA*	−10.0450818	Component of chemotactic signal transduction system
PA0414	*chpB*	−6.41019426	Probable methylesterase
PA0415	*chpC*	−3.29003495	Probable chemotaxis protein
PA0416	*chpD*	−4.43607137	Probable transcriptional regulator

### GSH Upregulates T3SS Expression Through Vfr

**Figure 3:**
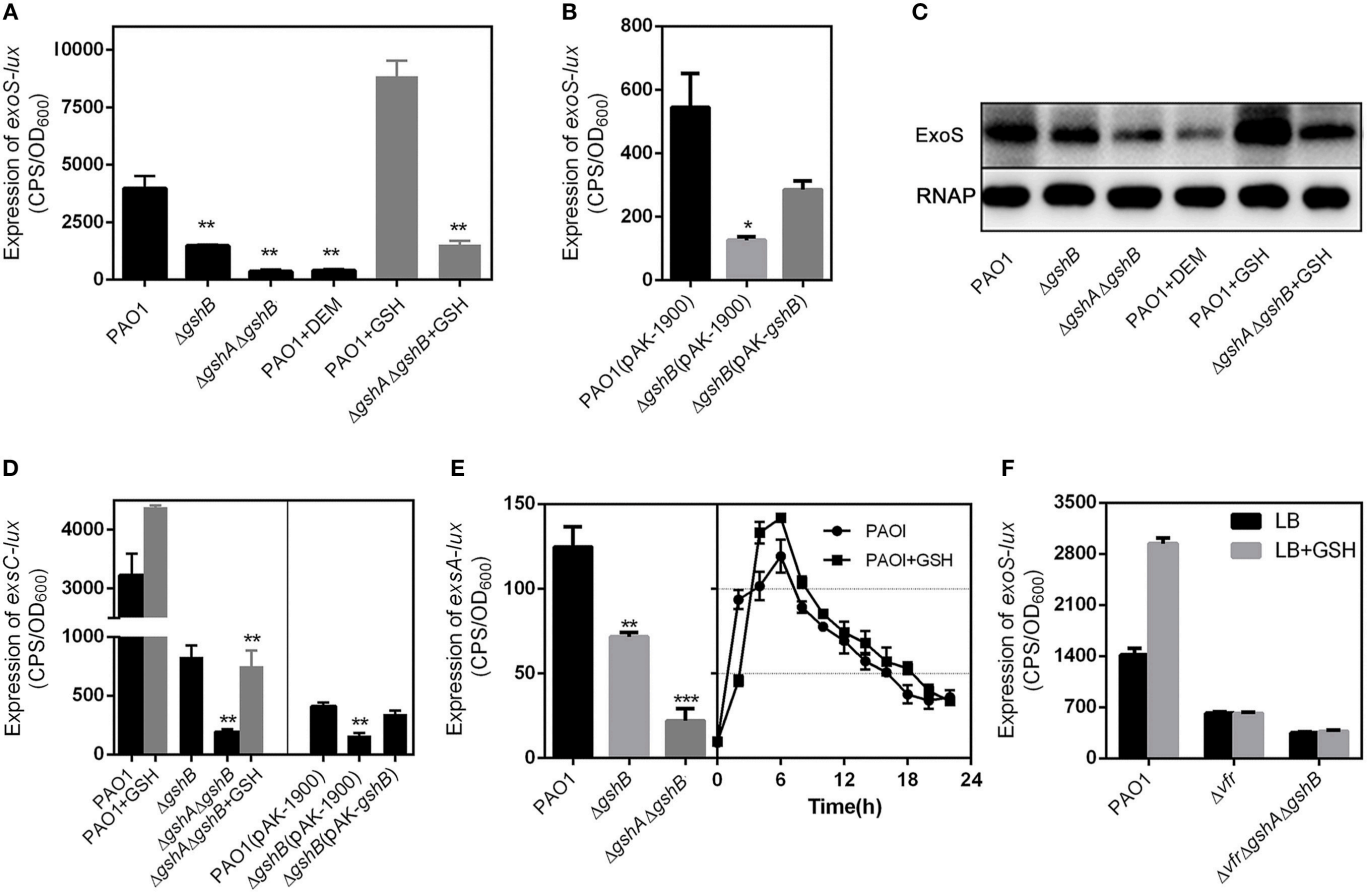
GSH upregulates the expression of T3SS genes through Vfr. All *P. aeruginosa* strains carrying plasmids, *exoS*-*lux*, P_*exsC*_*-lux*, and P_*exsA*_*-lux* were grown under T3SS inducing conditions (5 mM EGTA, 20 mM MgCl_2_) with or without GSH. The results shown are mean ± SEM. **(A)** The expression of *exoS* in the indicated *lux* reporter strains. **(B)** The expression of *exo*S in the genetically-complemented Δ*gshB* strain. **(C)** ExoS level in the indicated *P. aeruginosa* strains. Overnight bacterial cultures were subcultured in fresh media with or without 10 mM GSH for 3 h. Whole-cell extracts were resolved by SDS-PAGE and subjected to Western blotting. **(D)** The expression of P_*exsC*_ in PAO1, Δ*gshB*, Δ*gshAgshB* and genetically-complemented Δ*gshB* strains in the presence or absence of 10 mM GSH. **(E)** The expression of P_*exsA*_ in PAO1, Δ*gshB* and Δ*gshA*Δ*gshB*. **(F)** The expression of *exoS* was induced by GSH in Δ*vfr* and Δ*vfr*Δ*gshA*Δ*gshB*. *P < 0.05, **P < 0.01, ***P < 0.001, as determined by Student's *t*-test.

We monitored the expression of T3SS genes, *exoS* and *exoY*, under inducing conditions (LB broth supplemented with 5 mM EGTA and 20 mM MgCl_2_). Under low Ca^2+^ conditions, the transcription of *exoS* (Figure 3A) and *exoY* (Figure S2) genes were significantly reduced in both Δ*gshB* and Δ*gsh*AΔ*gshB* compared to wild-type PAO1. Additionally, the expression of *exoS* in Δ*gshA*Δ*gshB* was lower than that in Δ*gshB* (Figure 3A). The expression of *exoS* was partially restored in Δ*gshB* complemented with pAK-*gshB* (Figure 3B). To verify the involvement of GSH in T3SS expression, bacterial strains were treated with diethylmaleate (DEM) to deplete intracellular GSH. Significantly, the expression of *exoS* in DEM-treated PAO1 was reduced to levels comparable to that of untreated Δ*gshA*Δ*gshB* mutant (Figure 3A). In contrast, addition of GSH significantly increased the expression of *exoS* by 2-fold in wild-type PAO1 (Figure 3A). The amount of secreted ExoS mirrored that of mRNA expression (Figure 3C). These results indicate that GSH plays an important role in regulating the expression of *exoS*.

ExsA is the central regulator of T3SS gene expression. Previous studies have shown that EsxA binds to the promoters of T3SS genes and activates their transcription (Brutinel et al., 2008). *exsA* is co-transcribed with *exsC, exsE*, and *exsB* in the same operon under the control of the *exsC* promoter (P_*exsC*_) (Yahr and Frank, 1994). ExsA binds P_*exsC*_ promoter and autoregulates its own expression. A recent study has identified a second promoter (P_*exsA*_) located immediately upstream of *exsA* (Marsden et al., 2016). Thus, we examined whether the induction of *exoS* by GSH was under the control of both P_*exsC*_ and P_*exsA*_ promoter. To facilitate the analysis, P_*exsC*_-*lux* and P_*exsA*_-*lux* plasmid was introduced into both Δ*gshB* and Δ*gshA*Δ*gshB*. As expected, P_*exsC*_-*lux* and P_*exsA*_-*lux* reporter activity were drastically reduced when compared to PAO1 (Figures 3D,E). Genetic complementation of Δ*gshB* (Δ*gshB/gshB*) restored the expression of the *exsCEBA* operon. Importantly, the transcription of *exsCEBA* could be activated by GSH (Figure 3D). Because ExsA is an activator that feedback regulates its own operon, the loss of the *exsCEBA* transcription in the Δ*gshB* and Δ*gshA*Δ*gshB* mutants was likely caused by reduced *exsA* expression. These results suggest that GSH is directly or indirectly regulating the expression of the *exsCEBA* operon.

An earlier study has shown that Vfr directly activates *exsA* transcription by binding to the promoter P_exsA_ in *P. aeruginosa* (Marsden et al., 2016). Based on the aforementioned results, we speculated that the expression of T3SS modulated by GSH might be associated with Vfr. We generated a Δ*vfr* mutant to examine this hypothesis. As expected, provision of GSH did not alter the expression of *exoS* gene in Δ*vfr* (Figure 3F). Additionally, the Δ*vfr*Δ*gshA*Δ*gshB* triple mutant also failed to upregulate T3SS expression in the presence of exogenously provided GSH (Figure 3F). Importantly, the expression of *exoS* was significantly increased by GSH in Δ*vfr* complemented strain (Figure 5A). Collectively, these results suggest that GSH regulate the expression of TSSS gene through Vfr.

**Figure 4:**
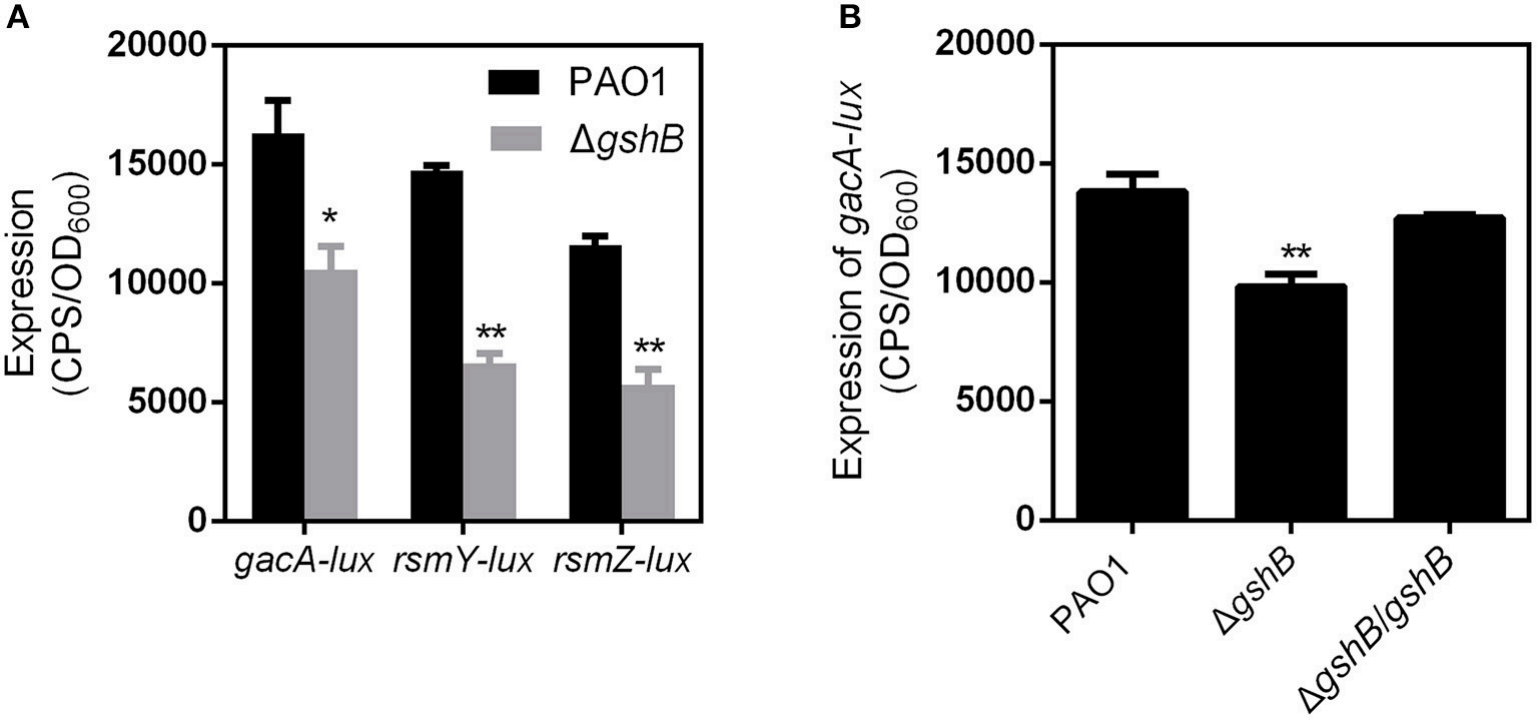
The expression of *vfr, gacA, rsmY, and rsmZ* genes in the indicated *P. aeruginosa* strains. **(A)** The expression of *gacA, rsmY, and rsmZ* genes in the PAO1 and Δ*gshB* strains. **(B)** The expression of *gacA* in the Δ*gshB* genetically-complemented strains. *P < 0.05, **P < 0.01 compared to PAO1 by Student's *t*-test.

In addition to Vfr and ExsA, the global transcriptional regulators RetS, LadS, and GacAS also regulate the expression of T3SS genes through the cAMP/Vfr pathway. RetS and LadS activities are mediated through the GacS/GacA/RsmZ. GacS activates the response regulator GacA by phosphotransfer. In turn, phosphorylated GacA positively regulates the expression of the two small RNAs, RsmY and RsmZ, which antagonize the activity of the regulator RNA-binding protein RsmA. Upregulation of *rsmY* and *rsmZ* and deletion of *rsmA* all result in the inhibition of the T3SS (Bordi et al., 2010). To clarify the relationship among GSH, GacS/GacA and T3SS, we examined the promoter activities of *gacS, gacA, rsmY*, and *rsmZ* in the Δ*gshB*. When compared to PAO1, a lower expression of *gacA, rsmY*, and *rsmZ* but not *gacS* was observed in Δ*gshB* and Δ*gshA*Δ*gshB* (Figure 4A). In addition, the expression of *gacA* could be restored to wild-type level in complemented strain Δ*gshB/gshB* (Figure 4B). These results indicated that the GSH upregulates the expression of GacA in *P. aeruginosa*. Due to contradictory finding in the regulation of GacA and GacS, more studies are needed to determine the impact of GSH on GacA/GacS-mediated expression of T3SS.

### Mutation of Cysteine Residues on Vfr Inhibits its Ability to Induce the Expression of T3SS Genes During Exposure to GSH

**Figure 5:**
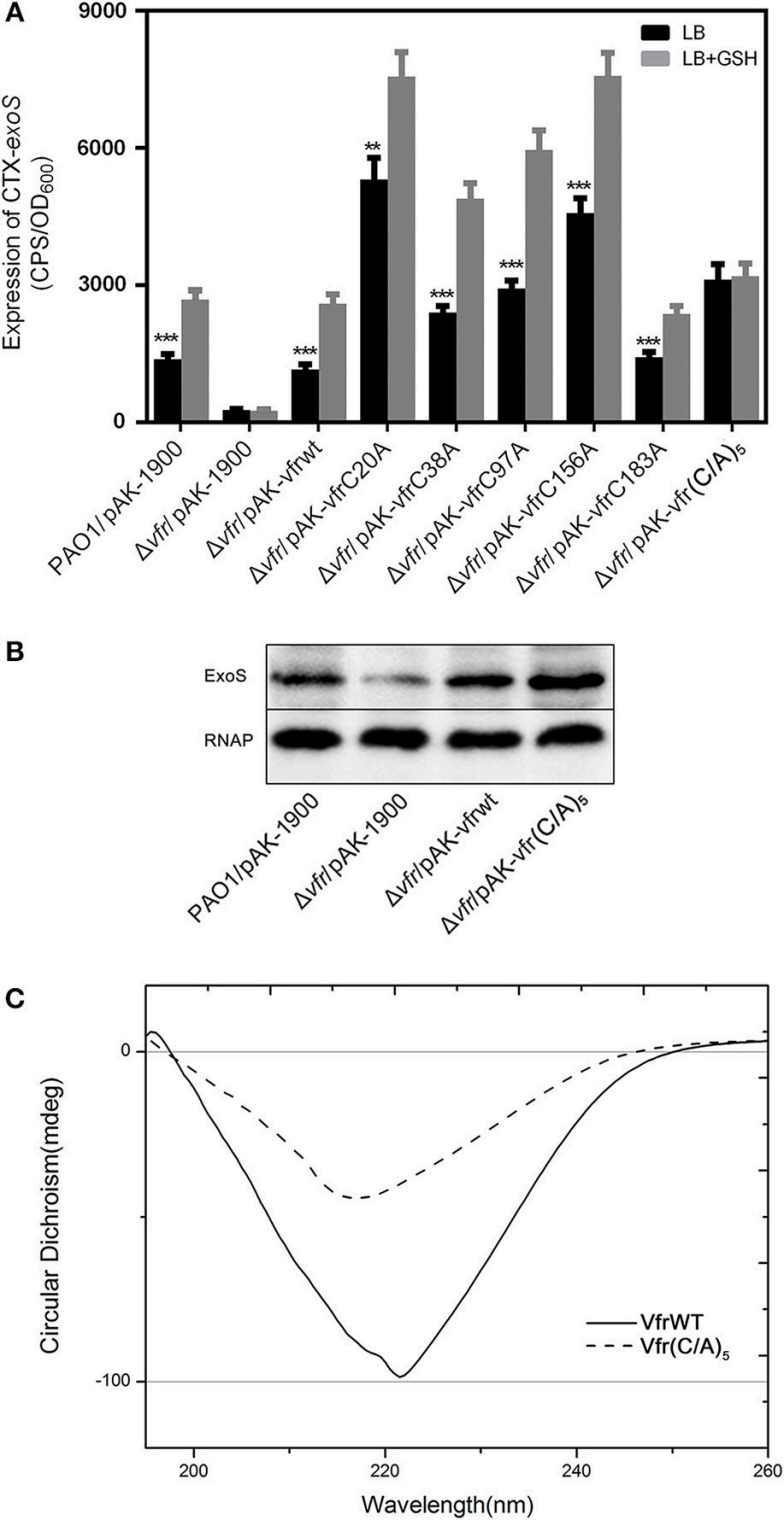
Cysteine residues in Vfr involved in signal transmission of glutathione. All *P. aeruginosa* strains carrying *exoS*-*lux* plasmids was grown under T3SS-inducing conditions (5 mM EGTA, 20 mM MgCl_2_) with or without 10 mM GSH. **(A)** Effect of cysteine-to-alanine mutation at positions 20, 38, 97, 156, and 183 of recombinant Vfr on the expression of *exoS*. **(B)** Secretion of ExoS in the indicated strains. **P < 0.01, ***P < 0.001 compared to PAO1 by Student's *t*-test. **(C)** Circular dichroism (CD) spectroscopy analysis of the secondary structure of Vfr and the Vfr (C/A)_5_.

Recently studies have suggested GSH can function as a cofactor for several enzymes. In *L. monocytogenes*, GSH is a cofactor of PfrA and increases its activity at target genes (Reniere et al., 2015). Additionally, VirA, the main regulator of T6SS1 in *Burkholderia pseudomallei*, upregualtes virulence when VirA dimers are converted to monomers in the presence of GSH (Wong et al., 2015). The cysteine residues on both PrfA and VirA proteins were important for proper functioning and sensing of GSH. For Vfr, the five cysteine residues are located on positions 20, 38, 97, 156, and 183. To determine whether cysteine residues in the Vfr of *P. aeruginosa* were directly involved in the sensing of intracellular GSH, we performed cysteine-to-alanine substitution on each cysteine on pAK-*vfr* by site-directed mutagenesis. The plasmids containing mutated variants of *vfr* were introduced into Δ*vfr* strain containing CTX-*exoS-lux*. Interestingly, all mutants have increased basal expression of *exoS* in the absence of exogenously supplied GSH, with VfrC20A and VfrC156A displayed the most significantly increase, followed by VfrC38A, VfrC97A, and VfrC183A (Figure 5A). Addition of GSH further enhanced *exoS* expression in all *vfr* mutants, suggesting all 5 cysteine residues were involved in GSH sensing (Figure 5A). Therefore, we generated a Vfr in which all five cysteine residues were mutated to alanine [Vfr(C/A)_5_]. Significantly, no increase in *exoS* expression was observed upon addition of GSH, indicating that intracellular GSH induced T3SS expression through Vfr (Figure 5A).

Because we observed higher expression and secretion of *exoS* when cysteine residues of Vfr were substituted with alanine (Figures 5A,B), we assessed whether these pheotypes could be attributed by the conformation change of Vfr(C/A)_5_. Circular dichroism (CD) experiments were performed to understand the secondary and tertiary structure characteristics of wild type Vfr and Vfr(C/A)_5_. The far CD profile of wild-type Vfr exhibited a typical shape corresponding to a α-helical rich secondary structure revealing a broad negative minimum at 222 nm (Figure 5C). In the Vfr(C/A)_5_, the peak showed a decrease in negative ellipticities of the CD spectrum, which was a characteristic of secondary structure conformation change, indicating that cysteine residues were important for Vfr native conformation. Hence, the increased *exoS* expression in Vfr(C/A)_5_ was likely due to the conformation change.

### Oxidized Vfr Is Reduced by GSH

**Figure 6:**
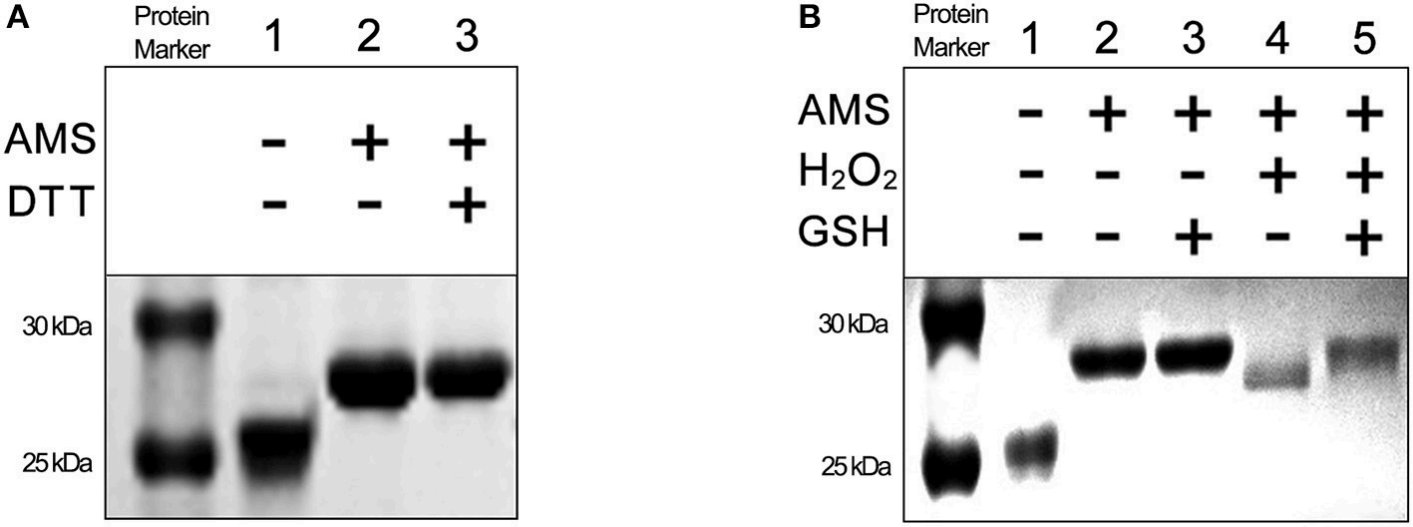
The redox state of cysteine thiol groups in Vfr under reducing and oxidizing conditions. Free thiol groups were covalently modified with AMS. Protein samples were resolved by using 15% non-reducing SDS-PAGE. **(A)** Vfr proteins (8 μM) treated with or without DTT (1 mM). **(B)** Vfr proteins (8 μM) treated with or without GSH (15 mM) and H_2_O_2_ (500 mM).

Cysteine residues on proteins are the most sensitive amino acid to oxidative modification. Also, they are important for protein conformation, function, stress response, and signal transduction inside the cell (Kalinina et al., 2014). Thiol-mediated redox control in cell metabolism is attributed to the ability of the cysteine-thiol to reversibly change its redox state that modulate changes of protein structure, catalytic and regulatory activities (Meyer and Hell, 2005). We suspected that the five cysteine residues on Vfr might be involved in thiol-mediated redox regulation of Vfr function. The redox state of cysteine-thiol in wild-type Vfr protein was examined by detection of free thiol groups using the AMS covalent modification method. As shown in Figure 6A, the migration of non-DTT-treated, AMS-modified Vfr (lane 2) were same as DTT-treated, AMS-modified Vfr (lane 3) on non-reducing sodium dodecyl sulfate polyacrylamide gel electrophoresis (SDS-PAGE), indicating that all cysteine residues on the wild-type Vfr were in the thiol state. To determine whether cysteine-thiol could be oxidized, the Vfr protein was incubated with and without H_2_O_2_ and analyzed by AMS modification on a non-reducing SDS-PAGE. As shown in Figure 6B, H_2_O_2_-treated, AMS-modified Vfr (lane 4) migrated faster than its untreated counterpart (lane 2), suggesting that there was a lack of thiol group after H_2_O_2_ exposure, and one or more cysteine-thiols might have been oxidized.

The cellular GSH is important for redox homeostasis and maintains the redox state of protein thiol groups (Meyer and Hell, 2005). We speculated that GSH might restore the oxidized form of Vfr to the reduced form. Following exposure to H_2_O_2_, oxidized Vfr proteins were treated with GSH. As expected, oxidized Vfr treated with GSH and modified with AMS (lane 5) had the same electrophoretic mobility as the untreated Vfr (lane 2) (Figure 6B). These results indicate that oxidized Vfr thiols can be restored to their reduced state in a reducing environment by GSH. Collectively, these data demonstrate that Vfr senses GSH-dependent redox environment to modulate the expression of T3SS genes.

## Discussion

We have previously shown GSH is important for the expression of *P. aeruginosa* T3SS genes. In this study, we examined the mechanism underlying the regulation of T3SS by GSH. Our results clearly demonstrate that GSH is a critical signaling molecule that regulates T3SS gene expression through the activation of Vfr. Vfr-deficient mutant fails to activate T3SS gene expression even in the presence of GSH, suggesting that it is a critical factor mediating signal transduction of GSH-mediated gene modulation. Our finding is further validated by the finding that when all cysteine residues of Vfr were substituted with alanine, the expression of T3SS could not be induced by GSH in Δ*vfr* [pAK-Vfr(C/A)_5_], suggesting these cysteine residues are directly involved in the sensing of GSH.

As a defensive measure, bacteria use redox sensitive transcription factors to sense and respond to oxidative stress (Lee et al., 2018). The thiol groups of cysteine residues in these proteins sense the redox state via reversible or irreversible modifications. Previous studies showed that several representative bacterial thiol-based redox-sensing proteins (VirA, OxyR, DksA, SpxA1, QsrA, OhrR/2-Cys, Spx, YodB, CrtJ, and CprK) regulate gene expression and play important roles during infection (Antelmann and Helmann, 2011; Wong et al., 2015). For example, the OxyR of *P. aeruginosa* senses cellular H_2_O_2_ level. High level of H_2_O_2_ cause cysteine residues in OxyR to form disulfide bonds and activate the transcription of antioxidant defense genes (Jo et al., 2015). However, when compared to OxyR, the VirA of *B. pseudomallei* works in opposite manner, where the oxidized dimers is reduced to its active monomeric form by GSH (Wong et al., 2015). GSH contributes to expression of *L. monocytogenes* virulence factors via allosteric binding to PrfA (Reniere et al., 2015). Similar to VirA, we propose that inactive, oxidized form of Vfr is converted to reduced active form in the presence of GSH. Therefore, Vfr in both Δ*gshB* and Δ*gshA*Δ*gshB* is in the oxidized state and therefore, is unable to self-activates *vfr* transcription because of inability to synthesize GSH. Provision of GSH to Δ*gshB* and Δ*gshA*Δ*gshB* restores Vfr activity through a reduction reaction that induces the expression of T3SS genes. Collectively, our results suggest that Vfr senses intracellular GSH levels to modulate the expression of ExsA and the downstream T3SS genes.

Our data show that the loss of *gshB* and *gshA* attenuates many virulence-associated phenotypes, including swimming, swarming and twitching motilities, biofilm formation, and virulence during acute pneumonia. RNA-seq analysis further corroborated that a number of genes, including *ppyR, migA, PilG*-*pilK, chpA-chpD*, associated with biofilm and motilities, were down regulated in Δ*gshA*Δ*gshB* (Attila et al., 2008; Lau et al., 2009). These phenotypes are consistent with a recently published study (Wongsaroj et al., 2018). Finally, in the course of our studies, we have also discovered that the expression of a subset of known T6SS genes are decreased in Δ*gshA*Δ*gshB*, suggesting that GSH also modulates the expression of T6SS system. However, the regulatory mechanism remains unclear.

In conclusion, the present work demonstrates that GSH represents a critical signaling molecule that activates the virulence of *P. aeruginosa*.

## Author Contributions

YaZ and HL: conception and design of the study, analysis, and interpretation of data, drafting the manuscript. CZ, XD, YuZ, WK, GC, GK, LY, and TW: acquisition of data and analysis of data. GL: critically revised the manuscript. All the authors participated the idea discussion and reviewed the manuscript.

### Conflict of Interest Statement

The authors declare that the research was conducted in the absence of any commercial or financial relationships that could be construed as a potential conflict of interest.
