# Supplementary material for: Glutathione Activates Type III Secretion System Through Vfr in Pseudomonas aeruginosa
Source: Front Cell Infect Microbiol. 2019 May 16;9:164. doi: 10.3389/fcimb.2019.00164 (PMC6532553; doi:10.3389/fcimb.2019.00164)
Supplement: Supplementary file 1 [file Table_1.docx]

**Supporting Information**

**Table S1. Bacterial strains and plasmids used in this study**

| Strains and plasmids | Related characteristics | Source |
| --- | --- | --- |
| ***E. coli*** |  |  |
| DH5a | *F^–^φ80lacZΔM15Δ(lacZYA-argF)U169 recA1 endA1 hsdR17(rk^–^,mk^+^) phoA supE44 thi-1 gyrA96 relA1 tonA* | Invitrogen |
| ***P. aeruginosa*** |  |  |
| PAO1 | Wild type | Lab stock |
| PA103 | Hypertoxigenic clinical isolate | Lab stock |
| Δ*gshA* | *gshA* deletion mutant of PAO1 | This study |
| Δ*gshB* | *gshB* mutant of PAO1; Gm^r^ | ([1](#_ENREF_1)) |
| Δ*gshA*Δ*gshB* | *gshA* and *gshB* double mutant of PAO1; Gm^r^ | This study |
| Δ*exsA* | *exsA* knockout mutant of PAO1; Gm^r^ | ([2](#_ENREF_2)) |
| Δ*vfr* | *vfr* mutant of PAO1; Gm^r^ | This study |
| Δ*gshA*Δ*gshB*Δ*vfr* | *gshA*, *gshB and vfr* knockout mutant of PAO1; Gm^r^ | This study |
| **Plasmids** |  |  |
| pEX18Tc | *oriT*^+^ *sacB*^+^ gene replacement vector with multiple-cloning site from pUC18; Tc^r^ | This study |
| pMS402 | Expression reporter plasmid carrying the promoterless *luxCDABE* gene; Kn^r^, Tmp^r^ | ([3](#_ENREF_3)) |
| pKD-*exoS* | pMS402 containing *exoS* promoter region; Kn^r^, Tmp^r^ | ([4](#_ENREF_4)) |
| pKD-*exoY* | pMS402 containing *exoY* promoter region; Kn^r^, Tmp^r^ | ([4](#_ENREF_4)) |
| pKD-P*exsA* | pMS402 containing *exsA* promoter region; Kn^r^, Tmp^r^ | ([5](#_ENREF_5)) |
| pKD-P*exsC* | pMS402 containing *exsC* promoter region; Kn^r^, Tmp^r^ | ([5](#_ENREF_5)) |
| pKD-*vfr* | pMS402 containing *vfr* promoter region; Kn^r^, Tmp^r^ | This study |
| CTX-*exoS-lux* | Integration plasmid, CTX6.1 with a fragment of pKD-*exoS* containing *exoS* promoter region and *luxCDABE* gene; Kn^r^, Tmp^r^, Tc^r^ | This lab |
| Mini-CTX1 | Gene delivery vector for inserting genes at the CTX phage *att* site on *P. aeruginosa* chromosome | ([6](#_ENREF_6)) |
| pAK-1900 | the *E. coli-P. aeruginosa* shuttle cloning vector carrying p*lac* upstream of MCS. Ap^r^, Cb^r^ | ([7](#_ENREF_7)) |
| pAK*-gshB* | pAK1900 with a 1207bp entire fragment of *gshB* between *Sal*I and *Hin*dIII; Ap^r^, Cb^r^ | This study |
| pAK*-gshA* | pAK1900 with a 1938bp entire fragment of *gshA* between *Sal*I and *Hin*dIII; Ap^r^, Cb^r^ | This study |
| pAK*-vfr* | pAK1900 with a 916bp entire fragment of *vfr* between *Sal*I and *Hin*dIII; Ap^r^, Cb^r^ | This study |
| *ptac-vfr* | pMMB containing *tacp*-*vfr*; *vfr* expression vector | This study |
| pAK*-vfrC5* | pAK1900 with a 916bp fragment of mutations *vfr* between *Sal*I and *Hin*dIII; Ap^r^, Cb^r^_._ Five cysteine residues in Vfr were mutated to alanine. | This study |

**Table S2. Primers used in this study**

| Primer | Application | Sequence |
| --- | --- | --- |
| *gshA*-Up-S | For constructing *gshA* deletion mutant | TCCAAGCTTGTCAAGGTCGCGGTGGTC |
| *gshA*-Up-A |  | AAGTCTAGAGCGGCGGGAGAGAAGATC |
| *gshA*-Down-S |  | ACATCTAGACTCCCTGGCCGAACAGAC |
| *gshA*-Down-A |  | AAGGAATTCGCCGCCGTAGAAGCTGAC |
| *gshB*-PAK-S | For *gshB*  complementation | ACTGGATCCTCATTGCGGATCGTGGTGT |
| *gshB*-PAK-A |  | ATCAAGCTTATCACGTCGCAACCGACC |
| *gshA*-PAK-S | For *gshA*  complementation | ATTGGATCCCAAGGCGACAAACTGGTC |
| *gshA*-PAK-A |  | TCGAAGCTTGGCCTCGCTGTACAACT |
| *vfr*-Up-S | For constructing *vfr* deletion mutant | TTAGAATTCCAGATGCGTATCGACGAG |
| *vfr*-Up-A |  | GAATCTAGACTGACGTACAAGGCGATG |
| *vfr*-Down-S |  | TTATCTAGAACCATGGTGGTCTTCGGC |
| *vfr*-Down-A |  | GTGAAGCTTGGAGCCCTCATTCGCATC |
| *vfr*-pAK-S | For constructing Δ*vfr* complementary strain | ATAGGATCCCGGACCGTCCATGACCAC |
| *vfr*-pAK-A |  | ATCAAGCTTCGCGCTGTCGAACCCCAT |
| *vfr*-promoter-S | For constructing *vfr* promoter | TTAGGATCCCGTTCAGACTCCCGCCGG |
| *vfr*-promoter-A |  | CGTTCAGACTCCCGCCGG |
| *vfr*-C20A-S | For constructing *vfr* C20 A mutant | CAAGCTGCTCGCACACGCTCACCGCCGCCGCTAC |
| *vfr*-C20A-A |  | GTAGCGGCGGCGGTGAGCGTGTGCGAGCAGCTTG |
| *vfr*-C38A-S | For constructing *vfr* C38A mutant | TATGCCGGCGATCGCGCCGAAACGCTGTTCTTCA |
| *vfr*-C38A-A |  | TGAAGAACAGCGTTTCGGCGCGATCGCCGGCATA |
| *vfr*-C97A-S | For constructing *vfr* C97A mutant | TTCGTGCCAAGGTGGAAGCCGAAGTCGCCGAGAT |
| *vfr*-C97A-A |  | ATCTCGGCGACTTCGGCTTCCACCTTGGCACGAA |
| *vfr*-C156A-S | For constructing *vfr* C156A mutant | ACCTTGCTGGACCTGGCCCAGCAACCGGACGCCA |
| *vfr*-C156A-A |  | TGGCGTCCGGTTGCTGGGCCAGGTCCAGCAAGGT |
| *vfr*-C183A-S | For constructing *vfr* C183A mutant | GAGATCGGCCGGATCGTCGGCGCCTCGCGGG |
| *vfr*-C183A-A |  | GCGCCGACGATCCGGCCGATCTCCTGGCGGG |

**Table S3. *P. aeruginosa* genes with altered mRNA levels in Δ*gshA*Δ*gshB* compared to the wild-type PAO1.**

| Gene ID | Gene name | Fold change | PseudoCAP Function Class |
| --- | --- | --- | --- |
| gene0010 | *tag* | 2.285766147 | DNA replication, recombination, modification and repair |
| gene0024 | *hemF* | -2.200484241 | Biosynthesis of cofactors, prosthetic groups and carriers |
| gene0025 | *aroE* | -2.04157016 | Amino acid biosynthesis and metabolism |
| gene0028 | PA0028 | -2.287111742 | Hypothetical, unclassified, unknown |
| gene0038 | PA0038 | -2.166227221 | Hypothetical, unclassified, unknown |
| gene0041 | PA0041 | -2.310210237 | Secreted Factors (toxins, enzymes, alginate) |
| gene0041a | PA0042 | -2.494663564 | hypothetical protein |
| gene0044 | *exoT* | -4.454667578 | Secreted Factors (toxins, enzymes, alginate) |
| gene0059 | *osmC* | -3.373826347 | Adaptation, Protection |
| gene0070 | PA0070 | -2.101948312 | Membrane proteins; Protein secretion/export apparatus |
| gene0072 | PA0072 | -2.140437651 | Membrane proteins; Protein secretion/export apparatus |
| gene0074 | *ppkA* | -2.118989176 | Adaptation, Protection; Translation, |
| gene0077 | *icmF1* | -2.010116816 | Protein secretion/export apparatus |
| gene0078 | PA0078 | -2.156860541 | Protein secretion/export apparatus |
| gene0083 | PA0083 | -2.065902151 | Protein secretion/export apparatus |
| gene0084 | PA0084 | -2.425912071 | Protein secretion/export apparatus |
| gene0085 | *hcp1* | -3.776572784 | Secreted Factors (toxins, enzymes, alginate) |
| gene0086 | PA0086 | -2.148343224 | Protein secretion/export apparatus |
| gene0087 | PA0087 | -2.517395508 | Protein secretion/export apparatus |
| gene0088 | PA0088 | -2.715542119 | Protein secretion/export apparatus |
| gene0089 | PA0089 | -3.157146499 | Protein secretion/export apparatus |
| gene0090 | *clpV1* | -3.681957917 | Protein secretion/export apparatus |
| gene0091 | *vgrG1* | -2.825759124 | Protein secretion/export apparatus |
| gene0096 | PA0096 | -2.415148358 | Hypothetical, unclassified, unknown |
| gene0097 | PA0097 | -3.132199466 | Hypothetical, unclassified, unknown |
| gene0098 | PA0098 | -3.602438567 | Hypothetical, unclassified, unknown |
| gene0099 | PA0099 | -3.115024209 | Hypothetical, unclassified, unknown |
| gene0100 | PA0100 | -2.301081374 | Hypothetical, unclassified, unknown |
| gene0101 | PA0101 | -2.721302451 | Hypothetical, unclassified, unknown |
| gene0103 | PA0103 | 2.007312205 | Membrane proteins; Transport of small molecules |
| gene0120 | PA0120 | -2.029852884 | Transcriptional regulators |
| gene0141 | PA0141 | -7.667206589 | Hypothetical, unclassified, unknown |
| gene0155 | *pcaR* | 2.087211299 | Carbon compound catabolism; Transcriptional regulators |
| gene0164 | PA0164 | 2.037557276 | Amino acid biosynthesis and metabolism |
| gene0165 | PA0165 | 2.665027103 | Hypothetical, unclassified, unknown |
| gene0197 | *tonB2* | -2.154206208 | Transport of small molecules |
| gene0198 | *exbB1* | -2.18291554 | Transport of small molecules |
| gene0199 | *exbD1* | -2.510845765 | Transport of small molecules |
| gene0200 | PA0200 | -2.490794163 | Hypothetical, unclassified, unknown |
| gene0202 | PA0202 | -2.357751188 | Putative enzymes |
| gene0263 | *hcpC* | -2.194455842 | Secreted Factors (toxins, enzymes, alginate) |
| gene0266 | *davT* | -2.271823898 | Delta-aminovalerate aminotransferase |
| gene0291 | *oprE* | -2.398262798 | Membrane proteins; Transport of small molecules |
| gene0293 | *aguB* | 2.134459215 | Amino acid biosynthesis and metabolism; Putative enzymes |
| gene0299 | *spuC* | -2.319406303 | Putative enzymes; Carbon compound catabolism |
| gene0310 | PA0310 | 2.074335178 | Hypothetical, unclassified, unknown |
| gene0315 | PA0315 | 3.6092685 | Hypothetical, unclassified, unknown |
| gene0329 | PA0329 | -2.35693597 | Hypothetical, unclassified, unknown |
| gene0330 | *rpiA* | 2.003453892 | Energy metabolism |
| gene0342 | *thyA* | 2.017420272 | Biosynthesis of cofactors, prosthetic groups and carriers; Nucleotide biosynthesis and metabolism |
| gene0354 | PA0354 | 2.179616715 | Hypothetical, unclassified, unknown |
| gene0355 | *pfpI* | -3.468229097 | Translation, post-translational modification, degradation |
| gene0356 | PA0356 | 2.168209248 | Hypothetical, unclassified, unknown |
| gene0363 | *coaD* | 2.979471059 | Central intermediary metabolism |
| gene0381 | *thiG* | 2.652030818 | Biosynthesis of cofactors, prosthetic groups and carriers |
| gene0382 | *micA* | 3.096183074 | DNA replication, recombination, modification and repair |
| gene0385 | *PA0385* | 3.003268096 | Hypothetical, unclassified, unknown |
| gene0389 | *PA0389* | 2.314219804 | Hypothetical, unclassified, unknown |
| gene0390 | *metX* | 2.602856925 | Amino acid biosynthesis and metabolism |
| gene0407 | *gshB* | -3.802290102 | Amino acid biosynthesis and metabolism; Biosynthesis of cofactors, prosthetic groups and carriers |
| gene0409 | *pilH* | -7.463788417 | Two-component regulatory systems; Chemotaxis; Motility & Attachment |
| gene0410 | *pilI* | -7.947971016 | Chemotaxis; Motility & Attachment |
| gene0411 | *pilJ* | -10.08630669 | Chemotaxis; Motility & Attachment |
| gene0412 | *pilK* | -9.582937278 | Chemotaxis; Motility & Attachment |
| gene0413 | *chpA* | -10.04508184 | Two-component regulatory systems; Motility & Attachment; Chemotaxis |
| gene0414 | *chpB* | -6.410194261 | Chemotaxis |
| gene0415 | *chpC* | -3.290034946 | Chemotaxis |
| gene0416 | *chpD* | -4.436071366 | Chemotaxis; Transcriptional regulators |
| gene0431 | PA0431 | 2.213875741 | Hypothetical, unclassified, unknown |
| gene0438 | *codB* | 2.083279058 | Nucleotide biosynthesis and metabolism; Transport of small molecules |
| gene0459 | PA0459 | -3.170841522 | Translation, post-translational modification, degradation |
| gene0460 | PA0460 | -3.094758859 | Hypothetical, unclassified, unknown |
| gene0492 | PA0492 | -4.179152073 | Hypothetical, unclassified, unknown |
| gene0493 | PA0493 | -3.733298958 | Putative enzymes |
| gene0494 | PA0494 | -4.516414764 | Putative enzymes |
| gene0495 | PA0495 | -3.081355949 | Hypothetical, unclassified, unknown |
| gene0496 | PA0496 | -3.694849119 | Hypothetical, unclassified, unknown |
| gene0498 | PA0498 | -2.321394512 | Hypothetical, unclassified, unknown |
| gene0500 | *bioB* | 4.374736551 | Biosynthesis of cofactors, prosthetic groups and carriers |
| gene0501 | *bioF* | 4.277896083 | Biosynthesis of cofactors, prosthetic groups and carriers |
| gene0502 | PA0502 | 3.53077283 | Biosynthesis of cofactors, prosthetic groups and carriers |
| gene0503 | PA0503 | 4.585085441 | Biosynthesis of cofactors, prosthetic groups and carriers |
| gene0504 | *bioD* | 3.918727401 | Biosynthesis of cofactors, prosthetic groups and carriers |
| gene0509 | *nirN* | -24.63930463 | Biosynthesis of cofactors, prosthetic groups and carriers; Energy metabolism |
| gene0510 | PA0510 | -35.08790368 | Biosynthesis of cofactors, prosthetic groups and carriers; Energy metabolism |
| gene0511 | *nirJ* | -21.80908646 | Biosynthesis of cofactors, prosthetic groups and carriers; Energy metabolism |
| gene0512 | PA0512 | -18.11093655 | Biosynthesis of cofactors, prosthetic groups and carriers; Hypothetical, unclassified, unknown; Energy metabolism |
| gene0513 | PA0513 | -17.92685414 | Biosynthesis of cofactors, prosthetic groups and carriers; Energy metabolism; Transcriptional regulators |
| gene0514 | *nirL* | -15.34641533 | Hypothetical, unclassified, unknown; Energy metabolism; Biosynthesis of cofactors, prosthetic groups and carriers |
| gene0515 | PA0515 | -18.10851685 | Biosynthesis of cofactors, prosthetic groups and carriers; Energy metabolism; Transcriptional regulators |
| gene0516 | *nirF* | -15.56079279 | Energy metabolism; Biosynthesis of cofactors, prosthetic groups and carriers |
| gene0517 | *nirC* | -13.41314159 | Biosynthesis of cofactors, prosthetic groups and carriers; Energy metabolism |
| gene0518 | *nirM* | -29.69233328 | Biosynthesis of cofactors, prosthetic groups and carriers; Energy metabolism |
| gene0519 | *nirS* | -22.27811445 | Energy metabolism |
| gene0520 | *nirQ* | -6.755774827 | Energy metabolism; Central intermediary metabolism |
| gene0521 | PA0521 | -9.868937477 | Energy metabolism |
| gene0522 | PA0522 | -4.632079555 | Hypothetical, unclassified, unknown |
| gene0523 | *norC* | -41.99936223 | Energy metabolism |
| gene0524 | *norB* | -38.70519167 | Energy metabolism |
| gene0525 | PA0525 | -11.96226919 | Energy metabolism |
| gene0526 | PA0526 | -12.5548437 | Hypothetical, unclassified, unknown |
| gene0543 | PA0543 | -2.2871899 | Hypothetical, unclassified, unknown |
| gene0545 | PA0545 | -2.088072571 | Putative enzymes |
| gene0546 | *metK* | 2.069953222 | Amino acid biosynthesis and metabolism; Central intermediary metabolism |
| gene0547 | PA0547 | 2.393451823 | Transcriptional regulators |
| gene0565 | PA0565 | 3.51463294 | Hypothetical, unclassified, unknown |
| gene0572 | PA0572 | -2.329260337 | Hypothetical, unclassified, unknown |
| gene0578 | PA0578 | 2.781509313 | Hypothetical, unclassified, unknown |
| gene0579 | *rpsU* | 3.625442294 | Translation, post-translational modification, degradation |
| gene0580 | *gcp* | 2.332139866 | Translation, post-translational modification, degradation |
| gene0583 | PA0583 | 2.04680908 | Biosynthesis of cofactors, prosthetic groups and carriers |
| gene0592 | *ksgA* | 2.140216687 | Transcription, RNA processing and degradation |
| gene0602 | PA0602 | -2.392639089 | Transport of small molecules |
| gene0603 | PA0603 | -4.619044445 | Transport of small molecules |
| gene0604 | PA0604 | -9.97468532 | Transport of small molecules |
| gene0605 | PA0605 | -4.839971534 | Membrane proteins; Transport of small molecules |
| gene0606 | PA0606 | -5.109772552 | Membrane proteins; Transport of small molecules |
| gene0607 | *rpe* | 2.171799555 | Energy metabolism |
| gene0608 | PA0608 | 2.036411879 | Carbon compound catabolism |
| gene0654 | *speD* | 4.414832328 | Central intermediary metabolism |
| gene0668.1 | PA0668.1 | -3.210586094 | Non-coding RNA gene |
| gene0705 | *migA* | 2.228218034 | Putative enzymes; Cell wall / LPS / capsule |
| gene0713 | PA0713 | -3.073992403 | Hypothetical, unclassified, unknown |
| gene0714 | PA0714 | -2.762237329 | Hypothetical, unclassified, unknown |
| gene0744 | PA0744 | -2.066275396 | Putative enzymes |
| gene0745 | PA0745 | -2.449688595 | Putative enzymes |
| gene0767 | *lepA* | 2.138182871 | Translation, post-translational modification, degradation; Protein secretion/export apparatus |
| gene0774 | PA0774 | 2.335575689 | Hypothetical, unclassified, unknown |
| gene0775 | PA0775 | 3.426524207 | Hypothetical, unclassified, unknown |
| gene0800 | PA0800 | -2.220967528 | Membrane proteins |
| gene0802 | PA0802 | 2.564061181 | Membrane proteins |
| gene0835 | *pta* | -3.580682983 | Carbon compound catabolism |
| gene0836 | *ackA* | -2.44045362 | Putative enzymes |
| gene0843 | *plcR* | -2.612463262 | Secreted Factors (toxins, enzymes, alginate) |
| gene0845 | PA0845 | -2.313924924 | Hypothetical, unclassified, unknown |
| gene0850 | PA0850 | 2.432381424 | Hypothetical, unclassified, unknown |
| gene0853 | PA0853 | -2.111644766 | Putative enzymes |
| gene0858 | PA0858 | 4.118683499 | Hypothetical, unclassified, unknown |
| gene0859 | PA0859 | 2.084830566 | Hypothetical, unclassified, unknown |
| gene0887 | *acsA* | -2.534196842 | Carbon compound catabolism; Central intermediary metabolism |
| gene0889 | *aotQ* | 2.302046361 | Membrane proteins; Transport of small molecules |
| gene0890 | *aotM* | 2.176015323 | Membrane proteins; Transport of small molecules |
| gene0914 | PA0914 | 2.038281348 | Hypothetical, unclassified, unknown |
| gene0915 | PA0915 | 6.399725814 | Hypothetical, unclassified, unknown |
| gene0916 | PA0916 | 3.949788203 | Hypothetical, unclassified, unknown |
| gene0918 | PA0918 | -3.748480836 | Energy metabolism |
| gene0945 | *purM* | 2.388282569 | Nucleotide biosynthesis and metabolism |
| gene0947 | PA0947 | 2.264801005 | Hypothetical, unclassified, unknown |
| gene0962 | PA0962 | -3.872450239 | Adaptation, Protection |
| gene1050 | PA1050 | -2.125075691 | Hypothetical, unclassified, unknown |
| gene1052 | PA1052 | -2.131088349 | Hypothetical, unclassified, unknown |
| gene1111 | PA1111 | -2.131842902 | Hypothetical, unclassified, unknown |
| gene1116 | PA1116 | 3.052089472 | Hypothetical, unclassified, unknown |
| gene1117 | PA1117 | 2.073701894 | Hypothetical, unclassified, unknown |
| gene1123 | PA1123 | -6.300058954 | Hypothetical, unclassified, unknown |
| gene1127 | PA1127 | -6.15340264 | Adaptation, Protection; Putative enzymes |
| gene1144 | PA1144 | -2.224352286 | Membrane proteins; Transport of small molecules |
| gene1157 | PA1157 | 2.102158854 | Transcriptional regulators; Two-component regulatory systems |
| gene1161 | *rrmA* | 2.321690084 | Transcription, RNA processing and degradation |
| gene1192 | PA1192 | 2.699546153 | Hypothetical, unclassified, unknown |
| gene1193 | PA1193 | 2.699782785 | Hypothetical, unclassified, unknown |
| gene1228 | PA1228 | 3.112492449 | Hypothetical, unclassified, unknown |
| gene1241 | PA1241 | 2.731671313 | Transcriptional regulators |
| gene1247 | *aph* | 2.085125686 | Antibiotic resistance and susceptibility |
| gene1247 | *aprE* | -2.220056383 | Secreted Factors (toxins, enzymes, alginate); Protein secretion/export apparatus |
| gene1299 | PA1299 | 2.438470818 | Hypothetical, unclassified, unknown |
| gene1300 | PA1300 | 3.141015669 | Transcriptional regulators |
| gene1305 | PA1305 | 2.539353745 | Hypothetical, unclassified, unknown; Membrane proteins |
| gene1317 | *cyoA* | 15.76378254 | Energy metabolism |
| gene1318 | *cyoB* | 12.27455981 | Energy metabolism |
| gene1319 | *cyoC* | 20.39523376 | Energy metabolism |
| gene1320 | *cyoD* | 12.51845162 | Energy metabolism |
| gene1321 | *cyoE* | 14.5355964 | Energy metabolism |
| gene1323 | PA1323 | -4.022855394 | Hypothetical, unclassified, unknown |
| gene1324 | PA1324 | -4.420374672 | Hypothetical, unclassified, unknown |
| gene1324.1 | PA1324.1 | -2.469830589 | Non-coding RNA gene |
| gene1337 | *ansB* | -2.262917695 | Amino acid biosynthesis and metabolism |
| gene1347 | PA1347 | -2.275816284 | Transcriptional regulators |
| gene1360 | PA1360 | 2.119843005 | Membrane proteins |
| gene1409 | *aphA* | 2.27204819 | Carbon compound catabolism |
| gene1410 | PA1410 | 2.204579458 | Transport of small molecules |
| gene1417 | PA1417 | -2.162956593 | Putative enzymes |
| gene1418 | PA1418 | -2.780305623 | Transport of small molecules |
| gene1422 | *gbuR* | 2.398437724 | Transcriptional regulators |
| gene1429 | PA1429 | -2.903570978 | Membrane proteins; Transport of small molecules |
| gene1471 | PA1471 | -2.240875963 | Hypothetical, unclassified, unknown |
| gene1475 | *ccmA* | 2.13328353 | Transport of small molecules |
| gene1486 | PA1486 | -2.686052227 | Hypothetical, unclassified, unknown |
| gene1504 | PA1504 | 2.489176976 | Transcriptional regulators |
| gene1508 | PA1508 | -2.596816204 | Hypothetical, unclassified, unknown |
| gene1509 | PA1509 | -2.015157653 | Hypothetical, unclassified, unknown |
| gene1523 | *xdhB* | -2.000091958 | Nucleotide biosynthesis and metabolism |
| gene1524 | *xdhA* | -2.162408086 | Nucleotide biosynthesis and metabolism |
| gene1537 | PA1537 | -2.042786133 | Putative enzymes |
| gene1543 | *apt* | 2.384392742 | Nucleotide biosynthesis and metabolism |
| gene1552 | *ccoP1* | 3.649176954 | Energy metabolism; Energy metabolism |
| gene1552.1 | *ccoQ1* | 3.874301045 | Energy metabolism |
| gene1553 | *ccoO1* | 3.689166725 | Energy metabolism; Energy metabolism |
| gene1554 | *ccoN1* | 4.671773001 | Energy metabolism; Energy metabolism |
| gene1555 | *ccoP2* | -9.077581758 | Energy metabolism; Energy metabolism |
| gene1555.1 | *ccoQ2* | -6.20125322 | Energy metabolism |
| gene1556 | *ccoO2* | -6.335531336 | Energy metabolism; Energy metabolism |
| gene1557 | *ccoN2* | -3.330185974 | Energy metabolism; Energy metabolism |
| gene1562 | *acnA* | -2.574021364 | Energy metabolism |
| gene1580 | *gltA* | 3.152525239 | Energy metabolism |
| gene1582 | *sdhD* | 2.339187373 | Energy metabolism |
| gene1585 | *sucA* | 2.017519493 | Amino acid biosynthesis and metabolism; Energy metabolism |
| gene1592 | PA1592 | -4.039841738 | Hypothetical, unclassified, unknown |
| gene1632 | *kdpF* | 4.836105469 | Transport of small molecules |
| gene1634 | *kdpB* | 2.127695126 | Transport of small molecules |
| gene1638 | PA1638 | 2.368191146 | Hypothetical, unclassified, unknown |
| gene1656 | PA1656 | -2.537790287 | Protein secretion/export apparatus |
| gene1657 | PA1657 | -6.218852909 | Protein secretion/export apparatus |
| gene1658 | PA1658 | -5.920727781 | Protein secretion/export apparatus |
| gene1659 | PA1659 | -4.774516242 | Protein secretion/export apparatus |
| gene1660 | PA1660 | -4.523601652 | Protein secretion/export apparatus |
| gene1661 | PA1661 | -4.142967458 | Protein secretion/export apparatus |
| gene1662 | PA1662 | -6.311739739 | Putative enzymes; Protein secretion/export apparatus |
| gene1663 | PA1663 | -4.616789704 | Transcriptional regulators; Protein secretion/export apparatus |
| gene1664 | PA1664 | -4.413763218 | Protein secretion/export apparatus |
| gene1665 | PA1665 | -5.62977334 | Protein secretion/export apparatus |
| gene1666 | PA1666 | -5.525133317 | Protein secretion/export apparatus |
| gene1667 | PA1667 | -3.883907402 | Protein secretion/export apparatus |
| gene1668 | PA1668 | -5.300389612 | Protein secretion/export apparatus |
| gene1669 | PA1669 | -4.846881697 | Membrane proteins; Protein secretion/export apparatus |
| gene1670 | *stp1* | -4.285882867 | Translation, post-translational modification, degradation; Protein secretion/export apparatus |
| gene1671 | *stk1* | -2.507374022 | Translation, post-translational modification, degradation; Protein secretion/export apparatus |
| gene1678 | PA1678 | 2.704161053 | Putative enzymes |
| gene1681 | *aroC* | 2.094847821 | Amino acid biosynthesis and metabolism |
| gene1682 | PA1682 | 2.083650789 | Membrane proteins; Transport of small molecules |
| gene1687 | *speE* | 2.624988455 | Amino acid biosynthesis and metabolism |
| gene1689 | PA1689 | 2.071591324 | Hypothetical, unclassified, unknown |
| gene1692 | PA1692 | -2.482885215 | Protein secretion/export apparatus |
| gene1694 | *pscQ* | -2.350353666 | Protein secretion/export apparatus |
| gene1695 | *pscP* | -2.196407364 | Protein secretion/export apparatus |
| gene1701 | PA1701 | -2.363138003 | Protein secretion/export apparatus |
| gene1703 | *pcrD* | -2.557732034 | Protein secretion/export apparatus |
| gene1704 | *pcrR* | -3.756506335 | Transcriptional regulators |
| gene1705 | *pcrG* | -3.181672126 | Protein secretion/export apparatus |
| gene1706 | *pcrV* | -3.384787644 | Protein secretion/export apparatus |
| gene1707 | *pcrH* | -5.928438036 | Secreted Factors (toxins, enzymes, alginate); Protein secretion/export apparatus |
| gene1708 | *popB* | -4.78755524 | Protein secretion/export apparatus |
| gene1709 | *popD* | -4.438461984 | Protein secretion/export apparatus |
| gene1710 | *exsC* | -2.757075954 | Translation, post-translational modification, degradation; Protein secretion/export apparatus |
| gene1711 | *exsE* | -4.088138121 | ExsE |
| gene1712 | *exsB* | -2.466005884 | Translation, post-translational modification, degradation; Protein secretion/export apparatus |
| gene1713 | *exsA* | -2.575630983 | Protein secretion/export apparatus; Transcriptional regulators |
| gene1714 | *exsD* | -2.201977245 | Hypothetical, unclassified, unknown |
| gene1715 | *pscB* | -2.263450077 | Protein secretion/export apparatus |
| gene1716 | *pscC* | -2.609245255 | Protein secretion/export apparatus |
| gene1717 | *pscD* | -3.023634465 | Protein secretion/export apparatus |
| gene1718 | *pscE* | -2.908510025 | Protein secretion/export apparatus; Chaperones & heat shock proteins |
| gene1719 | *pscF* | -2.567785177 | Protein secretion/export apparatus |
| gene1720 | *pscG* | -3.620911417 | Protein secretion/export apparatus; Chaperones & heat shock proteins |
| gene1721 | *pscH* | -2.277530197 | Protein secretion/export apparatus |
| gene1722 | *pscI* | -2.436681461 | Protein secretion/export apparatus |
| gene1723 | *pscJ* | -2.169155638 | Protein secretion/export apparatus |
| gene1746 | PA1746 | -4.58125457 | Hypothetical, unclassified, unknown |
| gene1747 | PA1747 | -2.73537061 | Hypothetical, unclassified, unknown |
| gene1750 | PA1750 | 2.030914788 | Amino acid biosynthesis and metabolism |
| gene1756 | *cysH* | 2.321704902 | Amino acid biosynthesis and metabolism |
| gene1757 | *thrH* | 2.888676684 | Amino acid biosynthesis and metabolism |
| gene1758 | *pabB* | 2.32988207 | Biosynthesis of cofactors, prosthetic groups and carriers |
| gene1768 | PA1768 | 2.318735361 | Hypothetical, unclassified, unknown |
| gene1771 | *estX* | 2.437387592 | Putative enzymes |
| gene1780 | *nirD* | -3.044537508 | Central intermediary metabolism |
| gene1789 | PA1789 | -2.89057736 | Hypothetical, unclassified, unknown |
| gene1790 | PA1790 | 2.132710182 | Hypothetical, unclassified, unknown |
| gene1791 | PA1791 | 2.338682585 | Hypothetical, unclassified, unknown |
| gene1796 | *folD* | 2.372894423 | Translation, post-translational modification, degradation; |
| gene1799 | *parR* | 2.298074105 | Transcriptional regulators; Two-component regulatory systems |
| gene1800 | *tig* | 2.061864071 | Cell division; Chaperones & heat shock proteins |
| gene1839 | PA1839 | 2.565104737 | Hypothetical, unclassified, unknown |
| gene1840 | PA1840 | 2.071966197 | Hypothetical, unclassified, unknown |
| gene1866 | PA1866 | 3.239375375 | hypothetical protein |
| gene1871 | *lasA* | -2.173592663 | Secreted Factors (toxins, enzymes, alginate); Translation, post-translational modification, degradation |
| gene1905 | *phzG2* | 2.321429834 | Secreted Factors (toxins, enzymes, alginate) |
| gene1909 | PA1909 | 3.217917598 | Hypothetical, unclassified, unknown |
| gene1913 | PA1913 | 2.504077739 | Hypothetical, unclassified, unknown |
| gene1920 | *nrdD* | -4.709807729 | Nucleotide biosynthesis and metabolism |
| gene1947 | *rbsA* | -3.159317159 | Transport of small molecules |
| gene1948 | *rbsC* | -2.423046023 | Transport of small molecules |
| gene1949 | *rbsR* | -2.060111226 | Carbon compound catabolism; Transcriptional regulators |
| gene1952 | PA1952 | -3.115198219 | Hypothetical, unclassified, unknown |
| gene1959 | *bacA* | 2.402704716 | Cell wall / LPS / capsule; Adaptation, Protection; Antibiotic resistance and susceptibility |
| gene1963 | PA1963 | 2.826065691 | Hypothetical, unclassified, unknown |
| gene1964 | PA1964 | 2.359669787 | Transport of small molecules |
| gene1965 | PA1965 | 2.030535106 | Hypothetical, unclassified, unknown |
| gene1994 | PA1994 | 2.017595324 | Hypothetical, unclassified, unknown |
| gene1999 | *dhcA* | 2.142864294 | Amino acid biosynthesis and metabolism; Carbon compound catabolism |
| gene2006 | PA2006 | 2.293833906 | Membrane proteins; Transport of small molecules |
| gene2007 | *maiA* | 2.698813032 | Carbon compound catabolism |
| gene2008 | *fahA* | 3.215092805 | Carbon compound catabolism |
| gene2009 | *hmgA* | 5.043810909 | Carbon compound catabolism |
| gene2010 | PA2010 | 2.716739195 | Transcriptional regulators |
| gene2012 | *liuD* | -2.212140803 | Carbon compound catabolism |
| gene2013 | *liuC* | -3.086125512 | Carbon compound catabolism |
| gene2014 | *liuB* | -2.497684149 | Carbon compound catabolism |
| gene2015 | *liuA* | -2.231589135 | Carbon compound catabolism |
| gene2018 | *mexH* | -2.183371801 | Transport of small molecules |
| gene2026 | PA2026 | 2.859826107 | Membrane proteins |
| gene2042 | PA2042 | 2.592647511 | Transport of small molecules |
| gene2056 | PA2056 | 3.297486082 | Transcriptional regulators |
| gene2063 | PA2063 | 2.480450626 | Hypothetical, unclassified, unknown |
| gene2074 | PA2074 | -2.64930987 | Hypothetical, unclassified, unknown |
| gene2116 | PA2116 | 2.12848635 | Hypothetical, unclassified, unknown |
| gene2119 | PA2119 | -2.281926336 | Putative enzymes |
| gene2125 | PA2125 | -4.27664596 | Putative enzymes |
| gene2126 | PA2126 | -5.387180711 | Transcriptional regulators |
| gene2127 | PA2127 | -2.845419421 | Transcriptional regulators |
| gene2136 | PA2136 | -4.255577499 | Hypothetical, unclassified, unknown |
| gene2154 | PA2154 | -2.396747085 | Membrane proteins |
| gene2157 | PA2157 | -2.48421168 | Hypothetical, unclassified, unknown |
| gene2159 | PA2159 | -2.215142821 | Hypothetical, unclassified, unknown |
| gene2160 | PA2160 | -2.259114099 | Putative enzymes |
| gene2165 | PA2165 | -2.182543148 | Energy metabolism |
| gene2169 | PA2169 | -2.982398551 | Hypothetical, unclassified, unknown |
| gene2176 | PA2176 | -2.657263981 | Hypothetical, unclassified, unknown |
| gene2177 | PA2177 | -2.031630922 | Two-component regulatory systems |
| gene2190 | PA2190 | -2.081497943 | Hypothetical, unclassified, unknown |
| gene2191 | *exoY* | -3.282481077 | Secreted Factors (toxins, enzymes, alginate) |
| gene2193 | *hcnA* | -7.487005574 | Central intermediary metabolism |
| gene2194 | *hcnB* | -7.970217506 | Central intermediary metabolism |
| gene2195 | *hcnC* | -12.75446542 | Central intermediary metabolism |
| gene2211 | PA2211 | -2.614614758 | Hypothetical, unclassified, unknown |
| gene2247 | *bkdA1* | -2.572527842 | Amino acid biosynthesis and metabolism |
| gene2248 | *bkdA2* | -2.564928664 | Amino acid biosynthesis and metabolism |
| gene2249 | *bkdB* | -2.631342268 | Amino acid biosynthesis and metabolism |
| gene2250 | *lpdV* | -2.302071681 | Amino acid biosynthesis and metabolism; Energy metabolism |
| gene2252 | PA2252 | 3.568551961 | Transport of small molecules |
| gene2253 | *ansA* | 2.161288293 | Amino acid biosynthesis and metabolism |
| gene2290 | *gcd* | 2.302038197 | Carbon compound catabolism; Energy metabolism |
| gene2291 | PA2291 | 2.034000506 | Transport of small molecules |
| gene2302 | *ambE* | -2.637367249 | Secreted Factors (toxins, enzymes, alginate); Putative enzymes |
| gene2303 | *ambD* | -2.563378462 | Secreted Factors (toxins, enzymes, alginate) |
| gene2304 | *ambC* | -2.689815849 | Secreted Factors (toxins, enzymes, alginate) |
| gene2322 | PA2322 | -2.26138804 | Transport of small molecules |
| gene2323 | PA2323 | -2.035103263 | Carbon compound catabolism |
| gene2327 | PA2327 | 2.10185622 | Membrane proteins; Transport of small molecules |
| gene2328 | PA2328 | 3.067814467 | Hypothetical, unclassified, unknown |
| gene2329 | PA2329 | 4.045619567 | Transport of small molecules |
| gene2330 | PA2330 | 3.799535753 | Hypothetical, unclassified, unknown |
| gene2331 | PA2331 | 5.812414037 | Membrane proteins |
| gene2352 | PA2352 | 2.4667421 | Putative enzymes |
| gene2381 | PA2381 | -3.558903108 | Hypothetical, unclassified, unknown |
| gene2413 | *pvdH* | -2.583790015 | Adaptation, Protection |
| gene2414 | PA2414 | -3.217679242 | Carbon compound catabolism |
| gene2415 | PA2415 | -2.576874566 | Membrane proteins |
| gene2435 | PA2435 | -2.037426218 | Membrane proteins; Transport of small molecules |
| gene2437 | PA2437 | -2.707573546 | Membrane proteins |
| gene2441 | PA2441 | 3.062903226 | Hypothetical, unclassified, unknown |
| gene2442 | *gcvT2* | 5.147434428 | Central intermediary metabolism; Amino acid biosynthesis and metabolism |
| gene2443 | *sdaA* | 4.034675514 | Amino acid biosynthesis and metabolism |
| gene2444 | *glyA2* | 6.382540467 | Amino acid biosynthesis and metabolism |
| gene2445 | *gcvP2* | 6.310030022 | Central intermediary metabolism; Amino acid biosynthesis and metabolism |
| gene2446 | *gcvH2* | 3.887777469 | Amino acid biosynthesis and metabolism |
| gene2459 | PA2459 | -2.185149765 | Hypothetical, unclassified, unknown |
| gene2486 | PA2486 | -2.106817765 | Hypothetical, unclassified, unknown |
| gene2513 | *antB* | -2.064077494 | Carbon compound catabolism |
| gene2538 | PA2538 | -2.15479918 | Membrane proteins |
| gene2557 | PA2557 | -2.461489751 | Fatty acid and phospholipid metabolism |
| gene2558 | PA2558 | 2.054339136 | Membrane proteins; Transport of small molecules |
| gene2560 | PA2560 | 2.143104164 | Hypothetical, unclassified, unknown |
| gene2562 | PA2562 | -2.323020372 | Hypothetical, unclassified, unknown |
| gene2579 | *kynA* | 2.631610655 | Putative enzymes |
| gene2606 | PA2606 | 2.56135072 | Hypothetical, unclassified, unknown |
| gene2608 | PA2608 | 2.133920662 | Hypothetical, unclassified, unknown |
| gene2619 | *infA* | 3.091690153 | Translation, post-translational modification, degradation |
| gene2629 | *purB* | 3.327790525 | Amino acid biosynthesis and metabolism; Nucleotide biosynthesis and metabolism |
| gene2637 | *nuoA* | 3.162123539 | Energy metabolism |
| gene2653 | PA2653 | 2.930138143 | Membrane proteins; Transport of small molecules |
| gene2660 | PA2660 | 2.413622682 | Hypothetical, unclassified, unknown |
| gene2662 | PA2662 | -5.643296985 | Membrane proteins |
| gene2663 | *ppyR* | -5.324527983 | Membrane proteins; Cell wall / LPS / capsule |
| gene2664 | *fhp* | -3.155240895 | Energy metabolism |
| gene2693 | PA2693 | 2.53382941 | Hypothetical, unclassified, unknown |
| gene2753 | PA2753 | -2.625614515 | Hypothetical, unclassified, unknown |
| gene2754 | PA2754 | -3.913576696 | Hypothetical, unclassified, unknown |
| gene2757 | PA2757 | 2.436635749 | Membrane proteins |
| gene2776 | PA2776 | -2.239213494 | Carbon compound catabolism |
| gene2780 | PA2780 | -2.885962483 | Hypothetical, unclassified, unknown |
| gene2781 | PA2781 | -3.239890946 | Hypothetical, unclassified, unknown |
| gene2783 | PA2783 | -2.11470292 | Hypothetical, unclassified, unknown |
| gene2787 | *cpg2* | -2.470333006 | Central intermediary metabolism |
| gene2788 | PA2788 | -2.589639342 | Adaptation, Protection; Chemotaxis |
| gene2795 | PA2795 | 2.070375128 | Hypothetical, unclassified, unknown |
| gene2798 | PA2798 | 2.056619245 | Transcriptional regulators; Two-component regulatory systems |
| gene2800 | PA2800 | 2.216609334 | Antibiotic resistance and susceptibility |
| gene2815 | PA2815 | -2.549282402 | Putative enzymes |
| gene2828 | PA2828 | 2.393313078 | Putative enzymes |
| gene2840 | PA2840 | 2.610566244 | Transcription, RNA processing and degradation |
| gene2845 | PA2845 | -2.002576854 | Hypothetical, unclassified, unknown |
| gene2851 | *efp* | 2.252115153 | Translation, post-translational modification, degradation |
| gene2860 | PA2860 | 2.316922336 | Hypothetical, unclassified, unknown |
| gene2888 | *atuC* | -2.064953779 | Putative enzymes |
| gene2889 | *atuD* | -2.247580632 | Putative enzymes |
| gene2890 | *atuE* | -2.758593743 | Putative enzymes |
| gene2911 | PA2911 | 2.090270433 | Membrane proteins; Transport of small molecules |
| gene2912 | PA2912 | 2.516764032 | Transport of small molecules |
| gene2913 | PA2913 | 2.226183724 | Transport of small molecules |
| gene2943 | PA2943 | 2.66876974 | Amino acid biosynthesis and metabolism |
| gene2950 | PA2950 | 2.185716585 | Protein secretion/export apparatus; Motility & Attachment |
| gene2965 | *fabF1* | 2.090355256 | Fatty acid and phospholipid metabolism |
| gene2969 | *plsX* | 2.248374425 | Fatty acid and phospholipid metabolism |
| gene3019 | PA3019 | 2.280324808 | Transport of small molecules |
| gene3030 | *mobA* | 2.309969287 | Biosynthesis of cofactors, prosthetic groups and carriers |
| gene3031 | PA3031 | -2.173455154 | Hypothetical, unclassified, unknown |
| gene3038 | PA3038 | -2.04059554 | Transport of small molecules |
| gene3040 | PA3040 | -2.981401854 | Hypothetical, unclassified, unknown |
| gene3041 | PA3041 | -2.595008737 | Membrane proteins |
| gene3042 | PA3042 | -3.520051689 | Hypothetical, unclassified, unknown |
| gene3049 | *rmf* | -2.24784531 | Translation, post-translational modification, degradation |
| gene3050 | *pyrD* | 2.107741259 | Nucleotide biosynthesis and metabolism |
| gene3054 | PA3054 | -3.893207295 | Hypothetical, unclassified, unknown |
| gene3091 | PA3091 | -2.100675864 | Hypothetical, unclassified, unknown |
| gene3136 | PA3136 | 2.253687045 | Transport of small molecules |
| gene3139 | PA3139 | 2.427298478 | Amino acid biosynthesis and metabolism; Putative enzymes |
| gene3162 | *rpsA* | 2.2555205 | Translation, post-translational modification, degradation |
| gene3179 | PA3179 | 3.490685792 | Hypothetical, unclassified, unknown |
| gene3234 | PA3234 | -2.789483594 | Membrane proteins; Transport of small molecules |
| gene3246 | *rluA* | 2.02232182 | Transcription, RNA processing and degradation |
| gene3283 | PA3283 | 2.100497526 | Hypothetical, unclassified, unknown |
| gene3284 | PA3284 | 2.320946232 | Hypothetical, unclassified, unknown |
| gene3307 | PA3307 | -2.064360147 | Hypothetical, unclassified, unknown |
| gene3308 | PA3308 | 2.287901321 | RNA helicase HepA |
| gene3309 | PA3309 | -4.447763135 | Hypothetical, unclassified, unknown |
| gene3327 | PA3327 | -2.730773484 | Adaptation, Protection |
| gene3328 | PA3328 | -2.420037396 | Putative enzymes |
| gene3329 | PA3329 | -2.324101958 | Hypothetical, unclassified, unknown |
| gene3330 | PA3330 | -2.977044432 | Putative enzymes |
| gene3331 | PA3331 | -2.414238798 | Adaptation, Protection; Carbon compound catabolism |
| gene3333 | *fabH2* | -2.493173492 | Fatty acid and phospholipid metabolism |
| gene3334 | PA3334 | -2.121534002 | Fatty acid and phospholipid metabolism |
| gene3335 | PA3335 | -2.555931274 | Hypothetical, unclassified, unknown |
| gene3336 | PA3336 | -2.677204142 | Membrane proteins; Transport of small molecules |
| gene3337 | *rfaD* | -2.155264795 | Cell wall / LPS / capsule |
| gene3388 | PA3388 | 2.216521444 | Hypothetical, unclassified, unknown |
| gene3391 | *nosR* | -17.11917861 | Membrane proteins; Energy metabolism |
| gene3392 | *nosZ* | -19.55294617 | Energy metabolism |
| gene3393 | *nosD* | -4.658956876 | Energy metabolism |
| gene3394 | *nosF* | -2.579378997 | Energy metabolism; Transport of small molecules |
| gene3436 | PA3436 | 2.716442123 | Hypothetical, unclassified, unknown |
| gene3458 | PA3458 | -2.676328769 | Transcriptional regulators |
| gene3465 | PA3465 | -2.237491331 | Membrane proteins |
| gene3480 | PA3480 | 2.284059634 | Nucleotide biosynthesis and metabolism |
| gene3489 | PA3489 | 2.038030705 | Membrane proteins |
| gene3514 | PA3514 | -2.025660415 | Transport of small molecules |
| gene3532 | PA3532 | 2.252959978 | Membrane proteins |
| gene3572 | PA3572 | -2.20775829 | Hypothetical, unclassified, unknown |
| gene3573 | PA3573 | -3.393731763 | Adaptation, Protection; Transport of small molecules |
| gene3608 | *potB* | 2.508101744 | Membrane proteins; Transport of small molecules |
| gene3609 | *potC* | 2.271742094 | Membrane proteins; Transport of small molecules |
| gene3610 | *potD* | 2.582706784 | Transport of small molecules |
| gene3612 | PA3612 | -3.035457929 | Hypothetical, unclassified, unknown |
| gene3613 | PA3613 | -3.972923356 | Hypothetical, unclassified, unknown |
| gene3614 | PA3614 | -3.199295431 | Hypothetical, unclassified, unknown |
| gene3615 | PA3615 | -2.648017586 | Hypothetical, unclassified, unknown |
| gene3633 | *ygbP* | 2.462328843 | Biosynthesis of cofactors, prosthetic groups and carriers |
| gene3641 | PA3641 | 3.394264498 | Transport of small molecules |
| gene3655 | *tsf* | 2.181177481 | Translation, post-translational modification, degradation |
| gene3656 | *rpsB* | 2.37778262 | Translation, post-translational modification, degradation |
| gene3683 | PA3683 | 2.538441263 | Hypothetical, unclassified, unknown |
| gene3691 | PA3691 | -4.195189949 | Hypothetical, unclassified, unknown |
| gene3700 | *lysS* | 2.098861787 | Amino acid biosynthesis and metabolism; Translation, post-translational modification, degradation |
| gene3701 | prfB | 2.16663693 | peptide chain release factor 1 |
| gene3712 | PA3712 | -2.234179118 | Hypothetical, unclassified, unknown |
| gene3713 | *spdH* | 2.556569979 | Hypothetical, unclassified, unknown |
| gene3726 | PA3726 | 2.0796295 | Hypothetical, unclassified, unknown |
| gene3727 | PA3727 | -2.361101201 | Hypothetical, unclassified, unknown |
| gene3728 | PA3728 | -2.14630066 | Hypothetical, unclassified, unknown |
| gene3729 | PA3729 | -2.271681688 | Hypothetical, unclassified, unknown |
| gene3733 | PA3733 | -3.026286757 | hypothetical protein |
| gene3741 | PA3741 | 2.341417327 | Hypothetical, unclassified, unknown |
| gene3770 | *guaB* | 2.07762404 | Nucleotide biosynthesis and metabolism |
| gene3788 | PA3788 | -2.134438172 | Membrane proteins |
| gene3789 | PA3789 | -2.034947245 | Membrane proteins |
| gene3790 | *oprC* | -3.466728697 | Transport of small molecules |
| gene3791 | PA3791 | -2.186170384 | Hypothetical, unclassified, unknown; Membrane proteins |
| gene3818 | PA3818 | 2.620809032 | Translation, post-translational modification, degradation; Adaptation, Protection |
| gene3819 | PA3819 | -3.145147995 | Membrane proteins |
| gene3823 | *tgt* | 2.362899868 | Transcription, RNA processing and degradation; Translation, post-translational modification, degradation |
| gene3824 | *queA* | 2.327853756 | Translation, post-translational modification, degradation |
| gene3828 | *lptF* | -4.625842306 | Membrane proteins; Transport of small molecules |
| gene3840 | PA3840 | -2.345169655 | Hypothetical, unclassified, unknown |
| gene3841 | *exoS* | -6.576079014 | Secreted Factors (toxins, enzymes, alginate) |
| gene3842 | *spcS* | -2.951518307 | SpcS |
| gene3844 | PA3844 | -3.35492908 | Hypothetical, unclassified, unknown |
| gene3860 | PA3860 | -2.05818594 | Putative enzymes |
| gene3870 | *moaA1* | -15.24916291 | Biosynthesis of cofactors, prosthetic groups and carriers |
| gene3871 | PA3871 | -37.60617066 | Translation, post-translational modification, degradation; Chaperones & heat shock proteins |
| gene3872 | *narI* | -45.38755345 | Energy metabolism |
| gene3873 | *narJ* | -66.67904713 | Energy metabolism |
| gene3874 | *narH* | -41.76646278 | Energy metabolism |
| gene3875 | *narG* | -71.60094105 | Energy metabolism |
| gene3876 | *narK2* | -45.36664887 | Membrane proteins; Transport of small molecules |
| gene3877 | *narK1* | -60.89834497 | Membrane proteins; Transport of small molecules |
| gene3888 | PA3888 | -3.17724733 | Membrane proteins; Transport of small molecules |
| gene3889 | PA3889 | -2.856470666 | Transport of small molecules |
| gene3890 | PA3890 | -2.966271766 | Membrane proteins; Transport of small molecules |
| gene3891 | PA3891 | -4.059991723 | Transport of small molecules |
| gene3892 | PA3892 | 3.639191954 | Hypothetical, unclassified, unknown |
| gene3893 | PA3893 | 2.486998431 | Membrane proteins |
| gene3894 | PA3894 | 2.820307629 | Membrane proteins |
| gene3910 | *eddA* | -2.001456165 | Nucleotide biosynthesis and metabolism; Secreted Factors (toxins, enzymes, alginate) |
| gene3911 | PA3911 | -11.74423455 | Hypothetical, unclassified, unknown |
| gene3912 | PA3912 | -8.844845294 | Hypothetical, unclassified, unknown |
| gene3913 | PA3913 | -7.059312924 | Putative enzymes |
| gene3914 | *moeA1* | -20.86023398 | Biosynthesis of cofactors, prosthetic groups and carriers |
| gene3914 | *mqoA* | 10.36366284 | Central intermediary metabolism; Energy metabolism |
| gene3915 | *moaB1* | -29.44410572 | Biosynthesis of cofactors, prosthetic groups and carriers |
| gene3919 | PA3919 | -2.349525342 | Hypothetical, unclassified, unknown |
| gene3922 | PA3922 | -2.11502883 | Hypothetical, unclassified, unknown |
| gene3953 | PA3953 | 2.358103328 | Hypothetical, unclassified, unknown |
| gene3966 | PA3966 | 2.878944655 | Membrane proteins |
| gene3967 | PA3967 | 7.348419364 | Hypothetical, unclassified, unknown |
| gene3979 | PA3979 | 2.974811586 | Hypothetical, unclassified, unknown |
| gene3980 | PA3980 | 2.257877444 | Hypothetical, unclassified, unknown |
| gene4067 | *oprG* | -3.047286123 | Membrane proteins |
| gene4118 | PA4118 | 2.102804618 | Hypothetical, unclassified, unknown |
| gene4129 | PA4129 | -2.4194699 | Hypothetical, unclassified, unknown |
| gene4130 | PA4130 | -2.419544376 | Central intermediary metabolism |
| gene4131 | PA4131 | -3.257938483 | Putative enzymes |
| gene4132 | PA4132 | -2.985155211 | Hypothetical, unclassified, unknown |
| gene4133 | PA4133 | -3.004236366 | Energy metabolism |
| gene4134 | PA4134 | -3.070023668 | Hypothetical, unclassified, unknown |
| gene4138 | *tyrS* | 2.392978319 | Amino acid biosynthesis and metabolism; Translation, post-translational modification, degradation |
| gene4159 | *fepB* | -2.169614915 | Transport of small molecules |
| gene4204 | *ppgL* | -2.848637007 | Hypothetical, unclassified, unknown |
| gene4207 | *mexI* | -2.557816022 | Membrane proteins; Transport of small molecules |
| gene4208 | *opmD* | -2.674033249 | Membrane proteins; Transport of small molecules |
| PA4235 | bfrA | -2.018286521 | Transport of small molecules; Adaptation, Protection |
| gene4236 | *katA* | -6.012292414 | Adaptation, Protection |
| gene4248 | *rplF* | 2.217090405 | Translation, post-translational modification, degradation |
| gene4267 | *rpsG* | 2.23266782 | Translation, post-translational modification, degradation |
| gene4268 | *rpsL* | 2.529733328 | Translation, post-translational modification, degradation |
| gene4275 | *nusG* | 2.167970403 | Transcription, RNA processing and degradation |
| gene4290 | PA4290 | -2.152284333 | Adaptation, Protection; Chemotaxis |
| gene4291 | PA4291 | 2.636124577 | Hypothetical, unclassified, unknown |
| gene4292 | PA4292 | 2.905984745 | Membrane proteins; Transport of small molecules |
| gene4311 | PA4311 | -2.084549986 | Hypothetical, unclassified, unknown |
| gene4334 | PA4334 | 2.681702279 | Membrane proteins; Transport of small molecules |
| gene4335 | PA4335 | 4.52074247 | Hypothetical, unclassified, unknown |
| gene4336 | PA4336 | 4.577369922 | Hypothetical, unclassified, unknown |
| gene4345 | PA4345 | -2.179845312 | Hypothetical, unclassified, unknown |
| gene4346 | PA4346 | -2.284793633 | Hypothetical, unclassified, unknown |
| gene4349 | PA4349 | -2.504946867 | Hypothetical, unclassified, unknown |
| gene4350 | PA4350 | -8.879027778 | Fatty acid and phospholipid metabolism |
| gene4351 | PA4351 | -8.009711274 | Fatty acid and phospholipid metabolism; Fatty acid and phospholipid metabolism |
| gene4352 | PA4352 | -6.351574362 | Hypothetical, unclassified, unknown |
| gene4431 | PA4431 | 2.438439978 | Putative enzymes |
| gene4432 | *rpsI* | 2.217425922 | Translation, post-translational modification, degradation |
| gene4433 | *rplM* | 2.755736359 | Translation, post-translational modification, degradation |
| gene4438 | PA4438 | 3.380719216 | Hypothetical, unclassified, unknown |
| gene4443 | *cysD* | 2.038620543 | Central intermediary metabolism; Amino acid biosynthesis and metabolism |
| gene4463 | PA4463 | -2.717488034 | Hypothetical, unclassified, unknown |
| gene4480 | *mreC* | 2.553628354 | Cell wall / LPS / capsule; Cell division |
| gene4481 | *mreB* | 2.059791851 | Cell wall / LPS / capsule; Cell division |
| gene4487 | PA4487 | -2.147415707 | Hypothetical, unclassified, unknown |
| gene4496 | PA4496 | -2.237676235 | Transport of small molecules |
| gene4519 | *speC* | 2.392702405 | Amino acid biosynthesis and metabolism |
| gene4525 | *pilA* | -2.34370846 | Motility & Attachment |
| gene4545 | *comL* | 2.672289187 | Cell wall / LPS / capsule |
| gene4550 | *fimU* | -2.00030426 | Motility & Attachment |
| gene4563 | *rpsT* | 3.502736454 | Central intermediary metabolism; Translation, post-translational modification, degradation |
| gene4568 | *rplU* | 2.253469324 | Translation, post-translational modification, degradation |
| gene4571 | PA4571 | -4.780366953 | Energy metabolism |
| gene4574 | PA4574 | 2.219523776 | Hypothetical, unclassified, unknown |
| gene4577 | PA4577 | -2.053630902 | Hypothetical, unclassified, unknown |
| gene4578 | PA4578 | -2.507726126 | Hypothetical, unclassified, unknown |
| gene4587 | *ccpR* | -16.00212741 | Energy metabolism |
| gene4602 | *glyA3* | 2.281363074 | Amino acid biosynthesis and metabolism |
| gene4607 | PA4607 | -2.015517806 | Hypothetical, unclassified, unknown |
| gene4610 | PA4610 | -2.503728557 | Hypothetical, unclassified, unknown |
| gene4611 | PA4611 | -3.119846855 | Hypothetical, unclassified, unknown |
| gene4628 | *lysP* | 2.342194964 | Membrane proteins; Transport of small molecules |
| gene4636 | PA4636 | 2.003709973 | Hypothetical, unclassified, unknown |
| gene4642 | PA4642 | 2.807440917 | Hypothetical, unclassified, unknown |
| gene4644 | PA4644 | 2.304393887 | Hypothetical, unclassified, unknown |
| gene4645 | PA4645 | 2.613633674 | Nucleotide biosynthesis and metabolism |
| gene4646 | *upp* | 2.500945434 | Nucleotide biosynthesis and metabolism |
| gene4670 | *prs* | 2.783837586 | Carbon compound catabolism; Nucleotide biosynthesis and metabolism |
| gene4672 | PA4672 | 2.187035244 | Translation, post-translational modification, degradation |
| gene4673 | PA4673 | 2.327602506 | Hypothetical, unclassified, unknown |
| gene4675 | PA4675 | 2.816133341 | Transport of small molecules |
| gene4676 | PA4676 | 2.090240084 | Putative enzymes |
| gene4678 | *rimI* | 2.072263976 | Translation, post-translational modification, degradation |
| gene4684 | PA4684 | 2.361290086 | Hypothetical, unclassified, unknown |
| gene4688 | *hitB* | 2.390513725 | Membrane proteins; Transport of small molecules |
| gene4693 | *pssA* | 2.128877725 | Fatty acid and phospholipid metabolism |
| gene4719 | PA4719 | 2.111962579 | Membrane proteins; Transport of small molecules |
| gene4720 | *trmA* | 2.334524873 | Transcription, RNA processing and degradation |
| gene4737 | PA4737 | -2.190046606 | Hypothetical, unclassified, unknown |
| gene4743 | *rbfA* | 2.009848438 | Translation, post-translational modification, degradation; Adaptation, Protection |
| gene4746 | PA4746 | 2.69799278 | Hypothetical, unclassified, unknown |
| gene4753 | PA4753 | 2.233804639 | Hypothetical, unclassified, unknown |
| gene4765 | *omlA* | 2.107388378 | Membrane proteins; Transport of small molecules |
| gene4770 | *lldP* | 3.296655247 | Transport of small molecules |
| gene4771 | *lldD* | 3.321512774 | Energy metabolism |
| gene4772 | PA4772 | 3.173222236 | Energy metabolism |
| gene4786 | PA4786 | -2.1061461 | Putative enzymes |
| gene4814 | *fadH2* | -2.221456616 | Fatty acid and phospholipid metabolism |
| gene4839 | *speA* | 2.392204648 | arginine decarboxylase |
| gene4840 | PA4840 | 3.163921687 | Hypothetical, unclassified, unknown |
| gene4851 | PA4851 | 2.230869901 | Hypothetical, unclassified, unknown |
| gene4852 | PA4852 | 2.077921878 | Hypothetical, unclassified, unknown |
| gene4853 | *fis* | 2.272307113 | DNA-binding protein Fis |
| gene4854 | *purH* | 2.566251614 | Nucleotide biosynthesis and metabolism |
| gene4868 | *ureC* | -2.162917613 | Central intermediary metabolism |
| gene4876 | *osmE* | -3.64426846 | Membrane proteins; Adaptation, Protection |
| gene4877 | PA4877 | -2.846449716 | Hypothetical, unclassified, unknown |
| gene4880 | PA4880 | -3.53766833 | Central intermediary metabolism |
| gene4890 | *desT* | 2.128922025 | Transcriptional regulators |
| gene4916 | PA4916 | -2.194934004 | Hypothetical, unclassified, unknown |
| gene4917 | PA4917 | -2.374316113 | Hypothetical, unclassified, unknown |
| gene4918 | PA4918 | -3.446281272 | Hypothetical, unclassified, unknown |
| gene4919 | *pncB1* | -3.758283874 | Biosynthesis of cofactors, prosthetic groups and carriers |
| gene4920 | *nadE* | -2.816089221 | Amino acid biosynthesis and metabolism; Biosynthesis of cofactors, prosthetic groups and carriers |
| gene4922 | *azu* | -5.607037235 | Energy metabolism |
| gene4928 | PA4928 | 2.581973598 | Hypothetical, unclassified, unknown |
| gene4934 | *rpsR* | 2.083435903 | Translation, post-translational modification, degradation |
| gene4935 | *rpsF* | 2.045367015 | Translation, post-translational modification, degradation |
| gene4985 | PA4985 | -2.283249694 | Hypothetical, unclassified, unknown |
| gene5016 | *aceF* | 2.095878336 | Carbon compound catabolism; Energy metabolism |
| gene5025 | *metY* | -3.139312607 | Amino acid biosynthesis and metabolism |
| gene5026 | PA5026 | -2.018505767 | Hypothetical, unclassified, unknown |
| gene5030 | PA5030 | 6.86209373 | Membrane proteins; Transport of small molecules |
| gene5033 | PA5033 | -2.507226989 | Hypothetical, unclassified, unknown |
| gene5049 | *rpmE* | 2.005019151 | Translation, post-translational modification, degradation |
| gene5060 | *phaF* | -2.305617377 | Central intermediary metabolism |
| gene5061 | PA5061 | -3.595716439 | Hypothetical, unclassified, unknown |
| gene5111 | *gloA3* | 4.501488241 | Central intermediary metabolism |
| gene5113 | PA5113 | -2.492120992 | Membrane proteins |
| gene5117 | *typA* | 2.047454438 | Adaptation, Protection |
| gene5118 | *thiI* | 2.898353663 | Biosynthesis of cofactors, prosthetic groups and carriers |
| gene5127 | PA5127 | 2.069362618 | Putative enzymes |
| gene5129 | *grx* | 2.135361768 | Energy metabolism; Nucleotide biosynthesis and metabolism |
| gene5130 | PA5130 | 2.77130744 | Hypothetical, unclassified, unknown |
| gene5171 | *arcA* | -6.221694261 | Amino acid biosynthesis and metabolism |
| gene5172 | *arcB* | -12.09723163 | Amino acid biosynthesis and metabolism |
| gene5173 | *arcC* | -16.03304247 | Amino acid biosynthesis and metabolism |
| gene5178 | PA5178 | -4.158428533 | Hypothetical, unclassified, unknown |
| gene5180 | PA5180 | 2.638462118 | Hypothetical, unclassified, unknown |
| gene5181 | PA5181 | 8.707023305 | Putative enzymes |
| gene5187 | PA5187 | -2.055341099 | Putative enzymes |
| gene5202 | PA5202 | 5.315905157 | Hypothetical, unclassified, unknown |
| gene5203 | *gshA* | -2.462330416 | Amino acid biosynthesis and metabolism; Biosynthesis of cofactors, prosthetic groups and carriers |
| gene5207 | PA5207 | -2.11759929 | Membrane proteins; Transport of small molecules |
| gene5212 | PA5212 | -2.926413357 | Hypothetical, unclassified, unknown |
| gene5230 | PA5230 | -4.239548152 | Membrane proteins; Transport of small molecules |
| gene5231 | PA5231 | -3.162257137 | Membrane proteins; Transport of small molecules |
| gene5274 | *rnk* | 2.047646928 | Transcriptional regulators |
| gene5286 | PA5286 | 2.183226703 | Hypothetical, unclassified, unknown |
| gene5289 | PA5289 | 2.58719299 | Hypothetical, unclassified, unknown |
| gene5298 | PA5298 | 3.171442219 | Nucleotide biosynthesis and metabolism |
| gene5316 | *rpmB* | 2.389871646 | Translation, post-translational modification, degradation |
| gene5321 | *dut* | 2.099895614 | Nucleotide biosynthesis and metabolism |
| gene5354 | *glcE* | -2.132312736 | Central intermediary metabolism; Carbon compound catabolism |
| gene5375 | *betT1* | 3.959154724 | Membrane proteins; Transport of small molecules |
| gene5406 | PA5406 | 2.071950483 | Hypothetical, unclassified, unknown |
| gene5424 | PA5424 | -3.809377742 | Membrane proteins |
| gene5426 | *purE* | 2.344677072 | Nucleotide biosynthesis and metabolism |
| gene5427 | *adhA* | -6.363971539 | Energy metabolism; Carbon compound catabolism |
| gene5429 | *aspA* | 2.856589014 | Amino acid biosynthesis and metabolism |
| gene5474 | PA5474 | -2.379418685 | Translation, post-translational modification, degradation |
| gene5475 | PA5475 | -2.922868498 | Hypothetical, unclassified, unknown |
| gene5479 | *gltP* | 2.263694101 | Membrane proteins; Transport of small molecules |
| gene5482 | PA5482 | -2.101935978 | Membrane proteins |
| gene5491 | PA5491 | 2.016418883 | Energy metabolism |
| gene5496 | *nrdJb* | -8.50646453 | Nucleotide biosynthesis and metabolism |
| gene5497 | *nrdJa* | -5.678950932 | Nucleotide biosynthesis and metabolism |
| gene5503 | PA5503 | 2.561892284 | Transport of small molecules |
| gene5530 | PA5530 | 19.76958497 | Membrane proteins; Transport of small molecules |
| gene5545 | PA5545 | -2.027013016 | Hypothetical, unclassified, unknown |
| gene5568 | PA5568 | 2.028597551 | Membrane proteins |
| gene5569 | *rnpA* | 2.111876834 | Translation, post-translational modification, degradation |
| gene5570 | *rpmH* | 2.304503447 | Central intermediary metabolism; Translation, post-translational modification, degradation |

**Table S4. T**he diameters of the zones of inhibition **for PAO1 and** **its derivative mutants to H_2_O_2_**

| Strians | PAO1 | ∆*gshB* | ∆*gshA*∆*gshB* | ∆*gshB*/gshB |
| --- | --- | --- | --- | --- |
| Diameter of zone of inhibition (mm) | 25.33±0.58 | 28.33±0.57 | 31.67±0.76 | 25.16±0.29 |

*For filter disk assays, bacterial were grown to the stationary phase in LB medium, inoculated in LB soft agar (0.5% agar), and cultivated for 30 min, then, sterile filter disks laid on top, and spotted with 5 µl of 30% H_2_O_2._ Plates were inoculated with overnight at 37℃. The diameters of the zones of inhibition was measured with a ruler. Data (means ± SEM) were collected from the three independent experiments*.

**
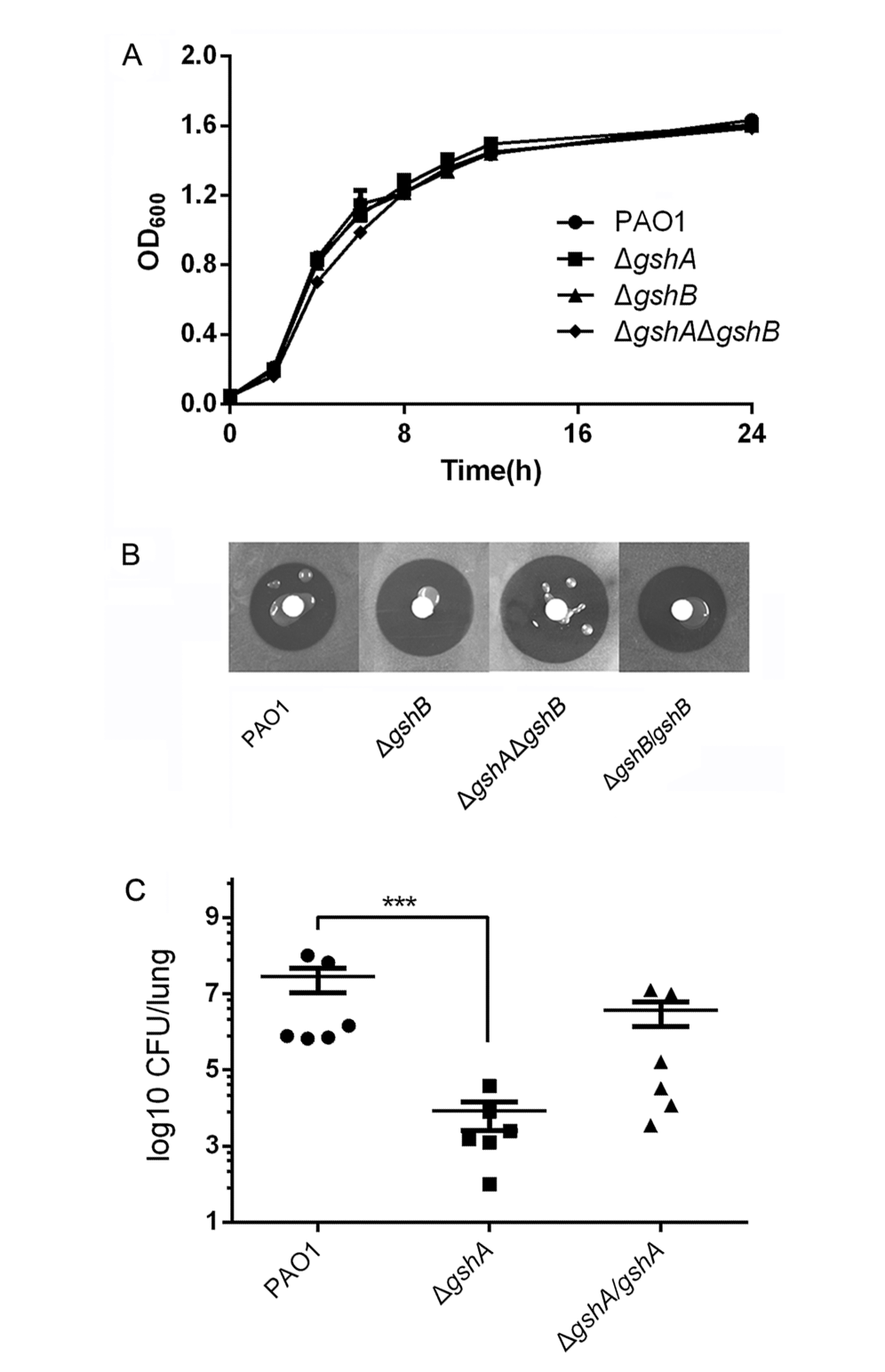
Figure S1. Sensitivity of *P. aeruginosa* strains to H_2_O_2_**. **(A)** Growth comparison between ∆*gshA*, ∆*gshB* and ∆*gshA*∆*gshB* to wild-type PA01 in LB broth. Bacterial growth (OD_600_) were measured for 24 h in a Victor^2^ Multilabel Counter (Perkin-Elmer). **(B)** Susceptibility of ∆*gshA*, ∆*gshB*, and ∆*gshA*∆*gshB* to H_2_O_2_ was compared to PAO1. For filter disk assays, bacterial were grown to the stationary phase in LB medium, inoculated in LB soft agar (0.5% agar), and cultured for 30 min. Then, sterile filter disks were placed on center of the agar, and spotted with 5 µl of 30% H_2_O_2。_ Similar results were obtained in more than three independent experiments. **(C)** Δ*gshA* was attenuated in acute pneumonia infection. 6-week old CD-1 mice (n=6) were intranasally infected with 1 x 10^7^ CFU of the indicated bacterial strains. Bacterial burden in infected lungs was assessed 16 h post-infection. Δ*gshA/gshA*: Δ*gshA* complemented with pAK-*gshA*. Data represent average CFU/lung ± standard deviations. ****p*＜0.001, when compared PAO1 to Δ*gshA*.


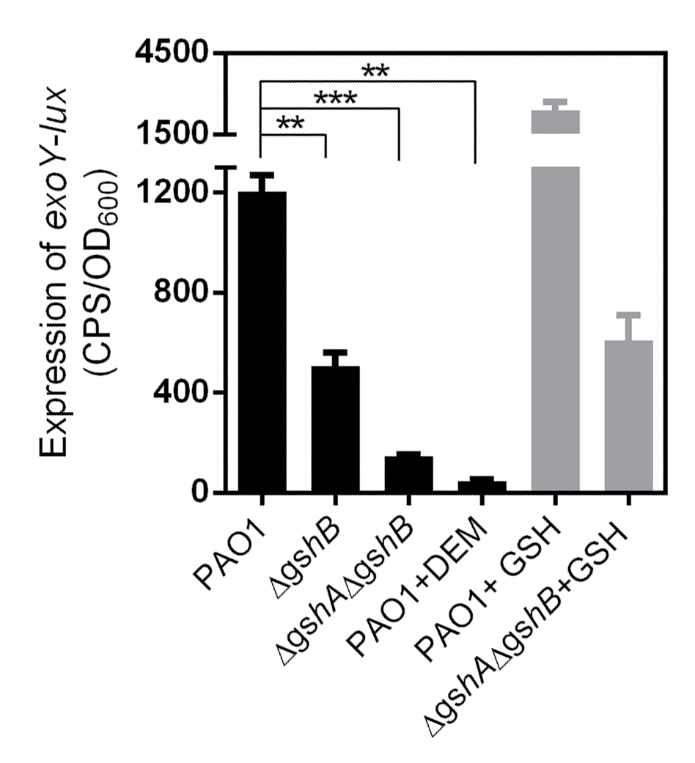


**Figure S2.** Glutathione (GSH) upregulates *exoY* expression. All bacterial strains were incubated in LB containing 5 mM EGTA and 20 mM MgCl_2_ with or without GSH. The results shown are mean ±sd.

**References**

1. Zhang, Y., and Duan, K. (2009) Glutathione exhibits antibacterial activity and increases tetracycline efficacy against *Pseudomonas aeruginosa*. *Science in China. Series C, Life sciences* **52**, 501-505

2. Kong, W., Chen, L., Zhao, J., Shen, T., Surette, M. G., Shen, L., and Duan, K. (2013) Hybrid sensor kinase PA1611 in *Pseudomonas aeruginosa* regulates transitions between acute and chronic infection through direct interaction with RetS. *Molecular microbiology* **88**, 784-797

3. Duan, K., Dammel, C., Stein, J., Rabin, H., and Surette, M. G. (2003) Modulation of *Pseudomonas aeruginosa* gene expression by host microflora through interspecies communication. *Molecular microbiology* **50**, 1477-1491

4. Duan, K., Dammel, C., Stein, J., Rabin, H., and Surette, M. G. (2003) Modulation of *Pseudomonas aeruginosa* gene expression by host microflora through interspecies communication. *Molecular microbiology* **50**, 1477-1491

5. Zhu, M., Zhao, J., Kang, H., Kong, W., and Liang, H. (2016) Modulation of Type III Secretion System in *Pseudomonas aeruginosa*: Involvement of the PA4857 Gene Product. *Frontiers in microbiology* **7**, 7

6. Hoang, T. T., Kutchma, A. J., Becher, A., and Schweizer, H. P. (2000) Integration-proficient plasmids for *Pseudomonas aeruginosa*: site-specific integration and use for engineering of reporter and expression strains. *Plasmid* **43**, 59-72

7. Poole, K., Neshat, S., Krebes, K., and Heinrichs, D. E. (1993) Cloning and nucleotide sequence analysis of the ferripyoverdine receptor gene *fpvA* of *Pseudomonas aeruginosa*. *Journal of bacteriology* **175**, 4597-4604
